# Isolation, Characterization, and Pathogenicity of Two *Pseudomonas syringae* Pathovars from *Populus trichocarpa* Seeds

**DOI:** 10.3390/microorganisms8081137

**Published:** 2020-07-28

**Authors:** Patricia MB Saint-Vincent, Mary Ridout, Nancy L. Engle, Travis J. Lawrence, Meredith L. Yeary, Timothy J. Tschaplinski, George Newcombe, Dale A. Pelletier

**Affiliations:** 1 Bioscience Division, Oak Ridge National Laboratory, Oak Ridge, TN 37831, USA; patricia.blair.saintvincent@gmail.com (P.M.S.-V.); englenl@ornl.gov (N.L.E.); lawrencetj@ornl.gov (T.J.L.); myeary16@comcast.net (M.L.Y.); tschaplinstj@ornl.gov (T.J.T.); 2 Geologic and Environmental Systems Directorate, National Energy Technology Laboratory, Pittsburgh, PA 15236, USA; 3 Department of Forest, Rangeland and Fire Sciences, University of Idaho, Moscow, ID 83844, USA; mridout@uidaho.edu (M.R.); georgen@uidaho.edu (G.N.)

**Keywords:** *Pseudomonas*, virulence, pathogen, metabolomics, germination

## Abstract

*Pseudomonas syringae* is a ubiquitous plant pathogen, infecting both woody and herbaceous plants and resulting in devastating agricultural crop losses. Characterized by a remarkable specificity for plant hosts, *P. syringae* pathovars utilize a number of virulence factors including the type III secretion system and effector proteins to elicit disease in a particular host species. Here, two *Pseudomonas syringae* strains were isolated from diseased *Populus* *trichocarpa* seeds. The pathovars were capable of inhibiting poplar seed germination and were selective for the *Populus* genus. Sequencing of the newly described organisms revealed similarity to phylogroup II pathogens and genomic regions associated with woody host-associated plant pathogens, as well as genes for specific virulence factors. The host response to infection, as revealed through metabolomics, is the induction of the stress response through the accumulation of higher-order salicylates. Combined with necrosis on leaf surfaces, the plant appears to quickly respond by isolating infected tissues and mounting an anti-inflammatory defense. This study improves our understanding of the initial host response to epiphytic pathogens in *Populus* and provides a new model system for studying the effects of a bacterial pathogen on a woody host plant in which both organisms are fully genetically sequenced.

## 1. Introduction

*Pseudomonas syringae*, a plant pathogen with at least sixty known host-specific pathovars, is one of the most broad-ranging and agriculturally relevant pathogens known [1]. Originally isolated in 1899 from diseased *Syringa vulgaris* (lilac), strains have been isolated from many economically and nutritionally important crops, including apples, beans, flowers, olives, peas, tomatoes, and rice. As a result of its ubiquity and simultaneous strain limitation, *P. syringae* is a useful model pathogen for studying plant host specificity as well as host immune response [2,3].

Infection and disease progression in the plant host occurs via the type III secretion system (T3SS), by which *P. syringae* translocates virulence factors into plant cells and affects transcription [4]. The T3SS is a common mechanism employed by both plant and animal pathogens, including members of *Erwinia*, *Escherichia*, *Pseudomonas*, *Salmonella*, and *Yersinia*, to name a few [5]. A complex system of secretion machinery, effector proteins, and chaperones, the T3SS genes are grouped together in a genomic region of the bacterial chromosome called the hypersensitivity response and pathogenicity (Hrp) island [6]. The T3SS effector proteins (TTEs), encoded by the avirulence (avr) genes, are secreted through the needlelike export system into the host cell cytosol and therein elicit the plant hypersensitivity response (HR) [7,8].

HR is the host’s attempt to isolate and inhibit the invading pathogen. Affected cells enter programmed cell death, resulting in both plant browning and soft rot; these necrotic lesions in turn limit resource acquisition by the pathogen and prevent the spread of the pathogen to other plant tissues. The model pathovar *P. syringae* pv. tomato DC3000 has been shown to infect not only its native host, but also the model herbaceous plant *Arabidopsis thaliana* [9]. In response to infection, *Arabidopsis* alters production of phenolic and indolic compounds, as well as sugars and amino acids. Thus, TTEs influence the plant metabolome in order to improve bacterial survival, even as the host plant mounts a defense response to eliminate the bacterial pathogen [4,10,11]. *P. syringae* has evolved to overcome host resistance through the acquisition and evolution of an impressive arsenal of TTEs as well as other virulence factors [4,12].

The suite of TTE genes harbored within an organism’s pathogenicity island does not directly correlate to host range, suggesting a more complicated and nuanced picture of host-pathogen communication [12,13]. TTEs may be responsible for infection of different plant surface such as the apoplast, leaf surface, or stem [14]. The acquisition of some TTEs may occur by horizontal gene transfer (HGT) while others have evolved over time within the species [15]. The *P. syringae* virulence strategy is not solely reliant on the T3SS; *P. syringae* pathovars are capable of producing phytoxins in addition to TTEs, including coronatine, phaseolotoxin, syringolin, syringomycin, syringopeptin, and tabtoxin [16]. These toxins, while not required for pathogenicity, can increase disease severity in the host but are metabolically intensive to produce [16]. Coronatine mimics the plant hormone methyl jasmonate in order to induce stress, while syringomycin and syringopeptin induce cell death through pore formation in the plasma membrane; tabtoxin inhibits glutamine synthetase and phaseolotoxin inhibits ornithine carbamoyltransferase [17,18]. The antimicrobial activity of these molecules may induce cell death of beneficial bacteria on the host plant, thereby enabling more successful pathogen colonization [16]. Gene clusters for the phytotoxins have been identified and are nonuniformly distributed among pathovars. Under standard laboratory conditions, many phytotoxins are not detectable, suggesting that the gene clusters are silent unless specific environmental conditions are met, such as the presence of competing organisms or host signaling molecules [2,16].

*P. syringae* pv. *aesculi*, a pathovar specific for *Aesculus hippocastanum* (European horse chestnut), groups with other woody host-associated bacteria when aligned based on whole genome sequencing [19]. Comparative genomics using these alignments reveals a region of the chromosome that putatively facilitates virulence in a woody host [20,21]. Termed the woody host and *Pseudomonas* species (WHOP) region, the cluster is composed of 14 open reading frames (ORFs) and includes genes for degradation of the phenolic monomers found in lignin [20,22,23]. While absent in all analyzed herbaceous pathovars, the WHOP region is also not essential for woody host pathogenicity and is absent from some woody host pathogens.

Unlike herbaceous plants, very few models exist for pathogen-woody plant systems, despite the heavy agricultural burden of this disease [22,24]. Model systems for *Olea europaea* (olive) [19,20], *Actinidia deliciosa* (kiwifruit) [20], *A. hippocastanum* (horse chestnut) [20], and *Prunus avium* (cherry) [25] have been developed, but the need for a better understanding of pathogen-woody plant infection will greatly benefit from a model system using a genetically tractable and well-studied plant species, such as *Populus*. Ubiquitous in North America but distributed globally, *Populus* spp. are not only important bioenergy crops but also a fully sequenced and genetically tractable model tree species [26]. *Pseudomonas syringae* infection of *Populus* species has been reported previously, often in association with significant frost damage resulting from the ice nucleating properties of the pathogen, which are thought to contribute to the dispersion of the pathogen [27,28,29]. A majority of unsequenced strains isolated from *P. tremuloides* induced a hypersensitive response in tobacco (95%, n = 21) and tomato (76%) plants [30]. Because of a well-studied *Populus* proteomic and metabolite response to various environmental stressors and individual bacteria [31,32], the development of a *Populus—P. syringae* model system will be beneficial in understanding the mechanics of woody host response to pathogenic bacteria.

Based on 16S rRNA gene sequencing, the organisms were determined to be members of the plant pathogen species *Pseudomonas syringae*. These pathogens are, to our knowledge, the first *P. syringae* pathovars from *Populus* to be isolated and subsequently genome-sequenced and characterized. We hypothesized that the *Populus* pathovars would be closely related to each other and specific to the host organism. Bioinformatic analysis of the genomes revealed a complete T3SS and TTEs similar to other phylogroup II isolates, multiple gene clusters responsible for toxin production, and the WHOP region in only one of the two isolates. Seed germination and leaf and plant assays were used to confirm pathogenicity and test host range. We then analyzed *P. trichocarpa* leaf tissue to study early host response to infection. The isolation and characterization of these tree pathogens sheds new light on plant-microbe interactions and enables plant-based experimentation to determine the effect of host genotype or microbiome composition on disease progression.

## 2. Materials and Methods

### 2.1. Sources for Bacteria, Plants, Seeds, and Chemicals

Unless otherwise noted, chemicals were purchased from Sigma-Aldrich (St. Louis, MO, USA). *P. syringae* pv. tomato DC3000 was acquired from ATCC (Manassas, VA, USA). *Populus deltoides* WV94, *Populus trichocarpa* BESC819, and *Populus trichocarpa* Nisqually-1 were acquired from Oak Ridge National Laboratory (Oak Ridge, TN, USA). *Arabidopsis thaliana* Col-0 seeds were purchased from Lehle Seeds (Round Rock, TX, USA). *Nicotiana tabacum* and *Spinacia oleracea* seeds were purchased from W. Atlee Burpee and Co (Warminster, PA, USA). *Phaseolus vulgaris* and *Populus tremuloides* seeds were purchased from Sheffield’s Seed Company (Locke, NY, USA). *Solanum lycopersicum* seeds were purchased from Johnny’s Selected Seeds (Waterville, ME, USA). *Triticum aestivum* seeds were purchased from Prairie Moon Nursery (Winona, MN, USA).

### 2.2. Seed Germination Assay

Open pollinated *Populus trichocarpa* seeds were dipped in sterile R2A media (control) or bacterial cultures adjusted to OD_600_ 0.5 in R2A for 5 min. The seeds were removed from the liquid and placed on sterile filter paper inside 10 cm Petri dishes. The treated seeds were incubated at 25 °C for 24 h prior to calculation of germination efficiency. The assay was repeated for three biological replicates of 50–100 seeds per treatment condition.

### 2.3. P. trichocarpa Seed Microbial Isolations and Growth Conditions

Mature *Populus trichocarpa* female catkins were collected on the University of Idaho campus, Moscow, ID, USA, in spring of 2017. Seeds were aseptically excised from 72 capsules and plated onto 4% potato dextrose agar PDA for a total of 1050 seeds (1 plate/capsule; 1–36 seeds/capsule, average of 14).

Seeds were incubated at 22 °C for eleven days to allow for germination and isolation of seed-associated microbes. Germination, microbial isolation frequency, and seedling mortality were recorded. Bacterial isolates with high isolation frequency were subcultured onto 4% PDA to pure culture. Bacterial isolates found associated with dead seedlings were streaked onto 4% PDA and nutrient broth agar (NBA). Strains NP10-3 and NP28-5 were selected for further analysis and genome sequencing. For maintenance, *P. syringae* strains were grown in R2A media (BD Difco, Franklin Lakes, NJ, USA) at 25 °C.

### 2.4. DNA Isolation and PCR

Genomic DNA was isolated from *Pseudomonas syringae* isolates grown to stationary phase in R2A media using the Wizard Genomic DNA Purification Kit (Promega Corp., Madison, WI, USA) according to the manufacturer’s instructions. Whole-genome sequencing was carried out at the U.S. Department of Energy (DOE) Joint Genome Institute (JGI) as described previously [33]. Draft genome sequences were generated using Illumina HiSeq2500-1TB technology and subjected to the JGI integrated microbial genomes database and comparative analysis workflow for assembly and annotation [34,35]. Draft genome sequences are publicly available in IMG (https://img.jgi.doe.gov). The genome sequences of *P. syringae* isolates were downloaded from the IMG database in December 2017.

### 2.5. Preparation of Sterile Filtered Culture Supernatants

Aliquots (10 mL) of sterile R2A media were inoculated with single colonies of *Pseudomonas* sp. NP10-3, *Pseudomonas* sp. NP28-5, or *P. syringae* pv. tomato DC3000 grown on R2A agar, and cultures were maintained at 25 °C with shaking for 24 h. Cells were pelleted and 2 mL of the supernatant was collected and passed through a sterile 0.2 µm filter (mdi Membrane Technologies, Inc., Harrisburg, PA, USA). The filtrate was air dried and resuspended in 100 µL sterile ddH_2_O.

### 2.6. Leaf Infection Assays

Leaves from 1 year old rooted cuttings from *P. trichocarpa* Nisqually-1 and *P. deltoides* WV94, maintained in a greenhouse, were cut using sterilized scissors and immediately submerged in sterile ddH_2_O. Leaves were then surface sterilized (5 min gentle shaking in 1% Tween-20 (*v*/*v* in sterile ddH_2_O), 1 min gentle mixing in 70% (*v*/*v*) EtOH, 10 min gentle shaking in 10% (*v*/*v*) NaOCl, followed by 3 rinses in sterile ddH_2_O) and placed on agar infused with 1× Hoagland’s No. 2 basal salt mixture and 1% glucose. Aseptically grown 3-month old *P. trichocarpa* BESC819 rooted cuttings were also harvested for leaves. Leaf surfaces were allowed to air dry in a biosafety cabinet and then wounded 3× or 6× per leaf with a sterile 25-gauge needle (Becton Dickinson, Franklin Lakes, NJ, USA). Stationary phase cultures of NP10-3, NP28-5, and DC3000 were diluted to OD_600_ 0.01 and 10-fold serially diluted in R2A media. Aliquots (10 µL) of bacterial cultures or media control were applied on top of each leaf wound. Plates were incubated at 25 °C and monitored every 12 h for infection. Experiments were carried out on three leaves for each of three biological replicates. High-resolution photos were analyzed using ImageJ to calculate coverage of infection.

The protocol was modified for *Arabidopsis* seedling infection. Seeds from *Arabidopsis*, wheat, and tomato were surface sterilized (5 min in 30% (*v*/*v*) NaOCl, 1 min in 0.1% Tween-20 in EtOH, 3 rinses in 100% EtOH and germinated on ½ × MS agar. *Arabidopsis* seeds were germinated in a growth chamber with a 16 h light/8 h dark photoperiod at 20 °C for 3 d prior to transferring to new ½ × MS agar plates infused with 0.1% sucrose. Stationary phase cultures of *P. syringae* isolates in R2A were spread along a line 1 cm below root tips and plates were incubated vertically in the same growth chamber for 2 weeks. High-resolution photos were analyzed using ImageJ to calculate root growth and branching.

Wheat and tomato seeds were surface sterilized as described for *Arabidopsis* except that the final 3 rinses were in sterile ddH_2_O. Seeds were germinated on wetted sterile filter paper at 22 °C for 3 d prior to transferring to agar plates infused with 1× Hoagland’s No. 2 basal salt mixture and 1% glucose. Seedlings were allowed to grow vertically for an additional 7 d in the growth chamber described for *Arabidopsis*. Then, leaves were wounded with a sterile 25-gauge needle and aliquots (10 µL) of bacterial cultures or media control, with the dilutions described above, were applied on top of each leaf wound. Plates were incubated at 25 °C and monitored every 12 h for infection.

Seeds from *P. tremuloides*, avocado, bean, pea, spinach, and tobacco plants were germinated in Miracle-Gro All Purpose Gardening Soil (Scotts Miracle-Gro, Marysville, OH, USA) and allowed to develop leaves (~1 month) in a growth chamber with 16 h light/8 h dark at 20 °C. Leaves were cut from plants, surface sterilized (5 min rinse in 1% Tween-20, 1 min rinse in 70% EtOH, 10 min rinse in 10% NaOCl, and 3 rinses in sterile ddH_2_O), and placed on agar plates infused with 1× Hoagland’s No. 2 basal salt mixture and 1% glucose. Leaves were wounded with a sterile 25-gauge needle challenged with bacteria as described above, with the following modification: bacterial cultures were diluted to OD_600_ 0.1 and tested in triplicate, but no dilutions were tested.

To test culture supernatants for lesion development, 3-month-old aseptically grown rooted cuttings of *P. trichocarpa* BESC819 were harvested for leaves, which were placed on agar plates infused with 1× Hoagland’s No. 2 basal salt mixture and 1% glucose. Leaves were wounded with a sterile 25-gauge needle 3× per leaf and treated with 10 µL aliquots of sterile ddH_2_O (negative control), resuspended culture supernatants from *P. syringae* isolates, or bacterial cultures at OD 0.001 (positive control). Plates were incubated at 25 °C and monitored every 12 h for infection.

### 2.7. Phylogenetic Tree

A concatenated alignment of 31 proteins (frr, pyrG, pgk, tsf, rpsE, rplD, rplK, infC, rplC, rplL, rplF, rplP, dnaG, rplA, rpsK, rplM, rplS, rpsJ, nusA, rpsI, rpsM, rpsS, rpsC, rplB, rplT, rplE, rpsB, rplN, smpB, rpmA, rpoB) for 22 *P. syringae* strains, including the newly sequenced *P.* sp. NP28-5 and P. sp. NP10-3 strains, and P. fluorescens Pf0-1 as an outgroup was generated and trimmed using the AMPHORA2 pipeline (Wu M, Scott AJ 2012) with HMMER v3.2.1 (hmmer.org). Alignment sites containing only gaps and ambiguous characters were removed using FAST v1.6 [36]. Molecular evolution model selection was performed using ModelFinder [37] comparing LG [38] empirical matrix models in combination with or without empirical profile mixture models C10-C60 [39], a proportion of invariant sites (+I), rate heterogeneity across sites using either a discrete gamma distribution with four rate categories (+4G) or the FreeRate model (+R) [40,41,42] with up to 32 categories, and empirical estimated amino acid frequencies (+F) using Bayesian Information Criterion. Phylogenetic analysis was conducted using maximum likelihood method implemented in IQ-TREE multicore v. 1.6.8 [43] using the LG + F + R2 model. Node support was evaluated using 1000 UFboot2 replicates [44,45]. The resulting phylogenetic tree was visualized and annotated using TreeGraph v. 2.15.0-887 beta (Stöver and Müller 2010).

### 2.8. Antibiotic Resistance Assay

R2A-agar plates were infused with sterile-filtered antibiotic solutions at the following final concentrations: ampicillin, 100 µg/mL; apramycin, 10 µg/mL; cefotaxime, 50 µg/mL; chloramphenicol, 50 µg/mL; gentamicin, 50 µg/mL; kanamycin, 50 µg/mL; nalidixic acid, 30 µg/mL; penicillin, 50 µg/mL; rifampicin, 50 µg/mL; tetracycline, 50 µg/mL. An amount of 5 mL R2A was inoculated with a single colony of *Pseudomonas* strains and grown to stationary phase with agitation at 25 °C. Sterile colony picking loops were used to spot ~2 µL stationary phase culture onto antibiotic-containing plates along 2 cm lines. Negative control was antibiotic-free R2A agar. Positive control strains were *Pseudomonas fluorescens* sp. GM30 and *Pseudomonas syringae* pv. tomato DC3000. Plates were incubated at 25 °C for 24 h and evaluated for growth.

### 2.9. Aromatic Compound Degradation Assay

The method for detection of anthranilate and catechol degradation was carried out as previously described [46]. Bacterial isolates were tested in biological triplicate and analyzed with two technical replicates. The compounds and degradation products were separated on an Agilent 1260 Infinity Quaternary LC (Agilent Technologies, Santa Clara, CA, USA) outfitted with an Eclipse Plus C_18_ column (4.6 mm ID × 250 mm, 5 µm particle size, Agilent Technologies) and using a flow rate of 1 mL/min and a 15 min gradient from 5–25% MeOH (0.1% “*v*/*v*” formic acid), monitoring 210 nm and 230 nm. Standard curves were generated for both anthranilate and catechol using known concentrations of each compound, prepared in triplicate.

### 2.10. IAA Production Assay

Single colonies of bacteria were used to inoculate M9 minimal media (MM) and grown to stationary phase with shaking at 25 °C. An aliquot (0.5 mL) was inoculated into 5 mL MM containing 200 mg/L L-Trp and grown to stationary phase with shaking at 25 °C. Strains were OD_600_-normalized prior to collection of 200 µL cell-free supernatant. The supernatant was mixed with 800 µL Salkowski’s reagent (300 mL conc. H_2_SO_4_, 500 mL ddH_2_O, 2.03 g FeCl_3_-6H_2_O) and allowed to react for 20 min at 22 °C prior to monitoring absorbance at 535 nm on a BioTek Synergy 2 multi-mode plate reader (BioTek U.S., Winooski, VT, USA). Values were normalized to control (sterile media) and a standard curve using known concentrations of indole-3-acetic acid (IAA) diluted into sterile media. IAA concentration was calculated using the following equation: [IAA] in µg/mL = (absorbance + 0.0018)/0.0694. Results are the average of six reads for each of 3 biological replicates.

### 2.11. Biofilm Formation Assay

The microtiter dish biofilm formation assay [47] was carried out as follows: bacterial strains (NP10-3, NP28-5, and DC3000) were grown in triplicate to stationary phase in M63 and Luria-Bertani (LB) broth (10% in PBS). Amounts of 100 µL aliquots were placed in triplicate in a 96-well plate and the cultures were incubated without shaking at 25 °C for 24 h. After 24 h, cultures were carefully shaken out. Wells were gently rinsed with sterile ddH_2_O 3× before a 15 min incubation with 125 µL 0.1% crystal violet solution. The plate was rinsed 3×, air-dried, and treated for 10 min with 125 µL of 30% (*v*/*v*) acetic acid in ddH_2_O. The solutions were transferred to a new microtiter plate and absorbance was monitored at 550 nm on a BioTek Synergy 2 multi-mode plate reader. A percentage of 30% acetic acid in ddH_2_O was used as the blank. *Pseudomonas aeruginosa* PA14 was used as a positive control for biofilm formation.

### 2.12. Motility Assay

Motility plates were prepared as described using LB agar plates (0.3% agar) [46]. Bacterial strains were grown to stationary phase in R2A liquid media, diluted into fresh media, and grown to OD_600_ 0.5. Plates were inoculated with 5 µL bacterial culture and bacterial growth was monitored over the course of 48 h. The appearance of a halo around the inoculation site was used as the indicator for bacterial motility. *Pseudomonas syringae* pv. tomato DC3000 was used as the positive control for motility.

### 2.13. Ice Nucleation Activity Assay

*P. syringae* strains were grown on King’s B media plates at 22 °C for 5 d. A single colony of *P. syringae* was suspended in 100 μL of potassium phosphate buffer (10 mM, pH 7) (PPB) by gentle vortexing. Then, 10 μL of this suspension was added to 2 mL of the same buffer prechilled in a −10 °C EtOH-ice water bath for 5 min. Strains were scored positive for ice nucleation activity if there was immediate ice formation in the tube. Results were confirmed by repeating in three biological replicates. *Pseudomonas syringae* pv. tomato DC3000 was used as the positive control for ice nucleation.

### 2.14. P. trichocarpa Metabolomics

*P. trichocarpa* genotype BESC819 shoot tips were sterilized by washing 5 min in 1% Tween-20, 1 min in 70% EtOH, 12 min in 0.6% NaOCl, and finally rinsed 3× in sterile ddH_2_O. Cuttings were rooted in tissue culture medium (1× MS salts (Caisson Laboratories, Logan, UT, USA), 0.5% activated charcoal (Sigma-Aldrich, St. Louis, MO, USA), 2% sucrose (VWR, Radnor, PA, USA), 0.05% MES (Sigma-Aldrich), 0.15% Gelrite (Plant Media, Dublin, OH, USA), and 0.1% PPM (Plant Cell Technology, Washington, DC, USA) for 3 weeks. Rooted cuttings were selected and planted in sterilized polycarbonate vessels (7.62 cm × 7.62 cm × 20.3 cm) containing 150 mL of autoclaved inert clay (Pro’s Choice Rapid Dry, Alpharetta, GA, USA) treated with 100 mL sterile Hoagland’s No. 2 basal salt mixture. Bacterial isolates were grown to stationary phase in R2A liquid media and diluted to 1 × 10^5^ cells/mL in 10 mM MgSO_4_. Sterile cotton swabs were used to treat the underside of each leaf of cuttings with bacterial isolates or 10 mM MgSO_4_. For the 0 h time point, bulk leaf tissue was immediately collected, flash frozen in N_2_ (l), and stored at −80 °C. Remaining plants were placed in a growth chamber with 12 h light (photosynthetically active radiation of 500 µmol m^−2^ s^−1^), 12 h dark, 22 °C, and 20% humidity. Bulk leaf tissue was harvested at 24 or 48 h from 3 plants at each time point for each treatment condition. One leaf from each plant was removed prior to flash freezing and used to confirm bacterial colonization. The leaf was placed on sterile R2A agar and incubated at 22 °C for 24 h, after which media was observed for the presence of bacterial colonies.

Leaf tissue was ground in N_2_ (l) and processed as previously described [48]. Briefly, 50 µg from each sample was extracted 2× overnight with 2.5 mL 80% EtOH (*v*/*v* in ddH_2_O) at 22 °C. Sorbitol (final concentration 10 ng/µL) was used as the internal standard to correct for extraction efficiency. An amount of 1 mL of pooled extract was dried with N_2_ (g), redissolved in MeCN, N-methyl-N-trimethylsilyltrifluoroacetamide with 1% trimethylchlorosilane and heated at 70 °C for 1 h to generate trimethylsilyl derivatives (TMS). Aliquots were injected into an Agilent 5975C inert XL gas chromatograph-mass spectrometer (Agilent Technologies, Santa Clara, CA, USA) under the following standard quadrupole gas chromatography mass spectrometry (GC-MS) conditions as previously described [49]: 70 eV electron impact ionization mode, targeting 2.5 full-spectrum (50 to 650 Da) scans s^−1^. Known metabolite peaks were extracted using a key characteristic *m*/*z* fragment and scaled to the total ion current with predetermined scaling factors. Peaks were quantified by area integration and concentrations were normalized using the sorbitol internal standard. Samples were analyzed in triplicate and compared against a user-created database of TMS-derivatized compounds from *P. trichocarpa*. *p*-value was calculated using the Student’s *t-*test. Fold change values were calculated relative to the 0 h time point for a particular treatment condition.

## 3. Results

### 3.1. Isolation of P. syringae from P. trichocarpa

**Figure 1:**
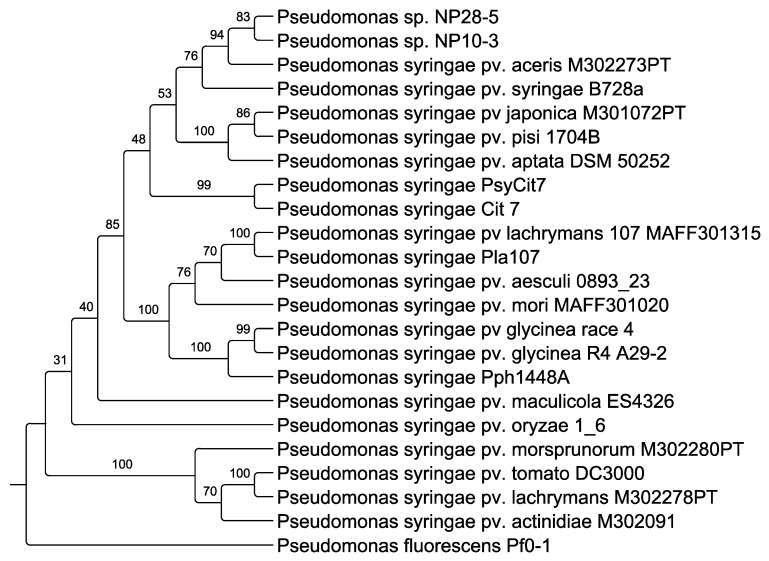
Maximum likelihood phylogenetic tree of amino acid sequences from 31 marker genes in *Pseudomonas syringae* pv. *populus* NP10-3 and NP28-5 (in bold) and selected representative *P. syringae*, with *P. fluorescens* Pf0-1 as out group. Branch lengths indicate expected substitutions per site and numbers above branches indicate ultrafast bootstrap support.

*Populus trichocarpa* female catkins that were infected with *Marssonnina* were aseptically dissected to remove the seeds. When cultivated on PD agar, bacterial outgrowth was observed from seeds which germinated but subsequently succumbed. Bacterial colonies from the dead seedling agar plates were picked and restreaked to isolate individual colonies. Two isolated colonies which were associated with dead seedlings were selected for further characterization. 16S rRNA gene sequencing and whole genome sequencing confirmed both isolates to be *Pseudomonas syringae* strains (Figure 1, Table 1). These *Populus* pathovars were subsequently named *Pseudomonas syringae* pv. *populus* NP10-3 and *Pseudomonas syringae* pv. *populus* NP28-5 (hereafter NP10-3 and NP28-5, respectively).

Because the *P. syringae* strains were isolated from *P. trichocarpa* seed capsules, we hypothesized that NP10-3 and NP28-5 were *Populus*-specific pathovars. In order to test this hypothesis, the strains would need to (1) exhibit pathogenic effects in *Populus* and (2) act in a species-specific manner. Pathogenic effects were studied in seed and seedling experiments for *Populus* species and in leaf-wounding experiments with other plants.

### 3.2. Effects on Germination of P. trichocarpa Seeds

**Figure 2:**
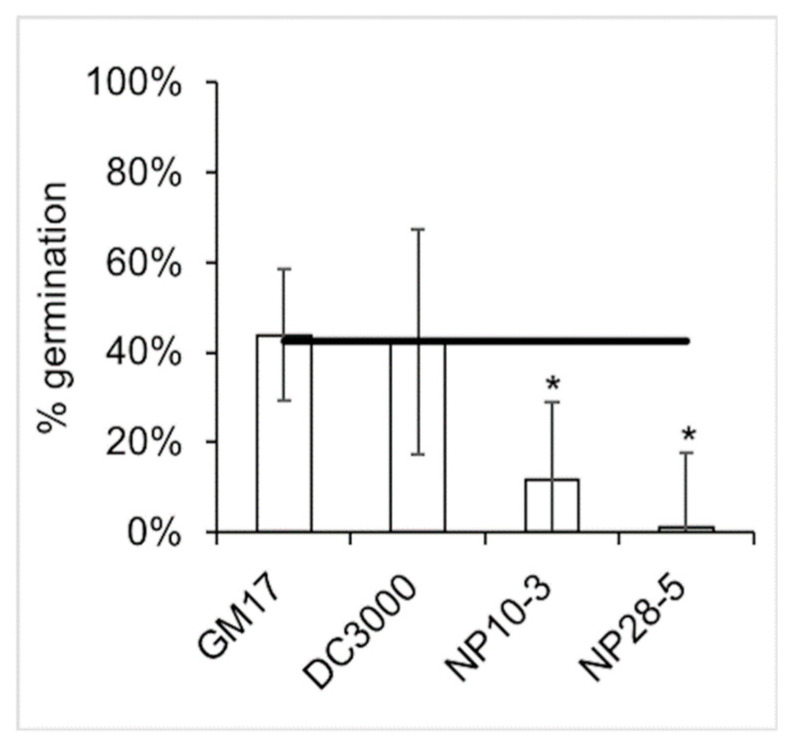
Germination efficiency of *P. trichocarpa* seeds treated with bacterial cultures. Horizontal line, control germination efficiency of seeds treated with sterile bacterial growth media. Error bars, standard deviation of three biological replicates of 50–100 seeds per treatment condition. *, *p* < 0.05.

First, we investigated the effects of the newly isolated and genome-sequenced strains on *Populus* seed germination. To this end, open pollinated *P. trichocarpa* seeds were soaked in sterile media or in bacterial cultures for 5 min and then placed on sterile filter paper inside 10 cm Petri dishes to incubate at 25 °C. Germination efficiency was calculated as the percentage of seeds that showed signs of germination after 24 h. NP10-3 and NP28-5 both decreased germination efficiency of the seeds compared to control (Figure 2). Plant growth-promoting bacterial strain *Pseudomonas* sp. GM17, isolated from the *P. deltoides* endosphere [33,50], did not significantly alter germination, nor did the *P. syringae* DC3000. The pathogens specific to *Populus* thus do affect the ability of seeds to germinate, unlike beneficial *Pseudomonas* isolates and tomato-specific *P. syringae*.

### 3.3. Genome Analysis

The DNA sequences of strains NP10-3 and NP28-5 assembled into genomes of 6,045,676 bp and 5,895,985 bp, respectively (Table 2). IMG/ER identified 5331 and 5116 genes within the genomes, respectively, with over 97% predicted protein coding density. Maximum likelihood phylogenetic analysis based on 31 marker genes of 20 representative *P. syringae* and *P. fluorescens* strains showed NP10-3 and NP28-5 form a cluster clade with other phylogroup II strains of *P. syringae* [22,34], and were found to be sisters to *P. syringae* pv. *aceris* M302273PT with strong bootstrap support (Figure 1). More broadly, the NP10-3 and NP28-5 sister strains are found within a clade containing *P. syringae* pv. *aceris* M302273PT, *P. syringae* pv. *syringae* B728a, *P. syringae* pv. *japonica* M301072PT, *P. syringae* pv. *pisi* 1704B, *P. syringae* pv. *aptata* DSM 50252, and *P. syringae* Cit7. Additionally, both *Populus* pathovars have an average nucleotide identity (ANI) of greater than 95% with *P. syringae* pv. *syringae* B728a (Table 2), and cluster with phylogroup II members of the *P. syringae* species, containing nearly all of the conserved phylogroup II-specific genes (Supplementary Table S1) [51]. In contrast to cherry tree isolates which are distributed across phylogroups (suggesting convergent evolution to pathogenicity), the *Populus* pathovars are highly related and cluster together [25,52].

*Pseudomonas syringae* is characterized by the presence of a set of conserved genes encoding a T3SS, and nearly one hundred TTEs have been identified and curated in the Hop Database [6,53]. Since the suite of effector proteins within the Hrp island of the genome contribute to, but do not fully explain, host specificity, we analyzed the sequenced genomes of NP10-3 and NP28-5 to gain further insight into their repertoire of potential pathogenicity factors. As with other phylogroup II members, NP10-3 and NP28-5 contain fewer TTEs than pathovars from other phylogroups (21 and 20 in each strain, respectively, Table S2). The two *Populus* pathovars share 18 TTEs, and NP10-3 shares 17 TTEs with *P. syringae* pv. *syringae* B728a (NP28-5 shares 16), but not *hopAB1*, which is considered the most important TTE related to apoplastic fitness in B728a [14]. Of the conserved TTEs, *hopH1*, *hopBE1 hopAP1*, and *hopAG1* have significantly different GC content compared to the overall genome, suggesting evolution of pathogenicity through HGT (Table S3). The Hop genes that differ between NP10-3 and NP28-5 (*hopBC1*, *hopAY1*, and *hopA2* in NP10-3 and *hopAF1* and *hopAL1* in NP28-5) all have divergent GC content compared to the entire genome, also suggesting HGT (Table S3).

Woody host-associated *Pseudomonas* (WHOP) regions in the chromosomes have been identified in many *Pseudomonas syringae* pathovars specific to woody hosts but are absent in pathovars specific to herbaceous plants [22]. Notably, not all woody host pathovars contain the WHOP region, including *P. syringae* pv. *aceris* ICMP 2802 (maple), *P. syringae* pv. *avellanae* ICMP 9746 (hazelnut), *P. syringae* pv. *avii* (cherry), *P. syringae* pv. *papulans* CFBP 1754 (apple), to name a few [46]. Surprisingly, only NP10-3 has a complete WHOP region; homologous open reading frames (ORFs) are absent in the slightly more virulent NP28-5 (Table 3). This suggests that the WHOP region is not required for virulence in *Populus* and that different mechanisms of pathogenicity are possible.

Catechol degradation is a common pathway in pathogenic bacteria, and although only NP10-3 contained an identifiable catechol catabolism cluster within the WHOP region, both isolates were able to grow in catechol-containing media (Figure S1A). Due to the low similarity of genes for anthranilate catabolism in the WHOP region (Table 3), we speculated that anthranilate would not be degraded by NP10-3. Indeed, when M9 minimal media was supplemented with anthranilate, a precursor in plant production of IAA, bacterial growth did not occur, nor was any degradation of anthranilate observed by HPLC analysis (Figure S1A).

Additionally, we profiled the antibiotic susceptibility of NP10-3 and NP28-5. Due to the presence of a ß-lactamase in the genomes of each organism, it is unsurprising that both were resistant to ampicillin and penicillin-G (Table S4). The high rate of ampicillin resistance in *P. syringae* suggests it is an ancestral trait, but resistance to other antibiotics is relatively rare [2].

### 3.4. Biosynthetic Potential of P. syringae Isolates

Secondary metabolites, also called natural products (NPs), are often used in chemical signaling in nature as antibiotics, antifungals, virulence factors, and more. *P. syringae* NPs include syringolin, syringomycin, and syringopeptin, which are nonribosomal peptide (NRP) and fused polyketide-NRP NPs implicated in pathogen virulence [54,55]. Using the biosynthetic gene cluster (BGC) identification programs PRISM and antiSMASH, the annotated genomes of NP10-3 and NP28-5 were mined for the presence of BGCs responsible for the production of the five main *P. syringae* toxins. Both NP10-3 and NP28-5 harbor phaseolotoxin, coronatine, syringolin, and syringomycin gene clusters, but not a tabtoxin gene cluster (Figure S2A). However, under the conditions tested, only coronatine and syringolin could be identified in culture supernatants (Figure S2B–G). Certain environmental cues or plant-produced signaling molecules may be required to induce production of metabolically costly molecules such as these toxins [14]. Indeed, virulence in *P. syringae* has been linked to the ability of the pathogen to produce and detect quorum sensing acyl-homoserine lactone (AHL) signaling molecules [56], and AhlI/R homologs are present in both NP10-3 and NP28-5. NP10-3 and NP28-5 may induce production of these toxins in response to the presence of *Populus* metabolites or the host immune response.

Additional NP BGCs were identified, including those for ectoine, a siderophore, and an arylpolyene (Figure S2A). 3-methylarginine, biosynthesized by an *S*-adenosylmethionine-dependent methyltransferase, was found in the exudate of *P. syringae* pv. *syringae* Pss22d and inhibited the growth of *P. syringae* pv. *Glycinea* [57]; the presence of its gene cluster suggests that NP10-3 and NP28-5 are equipped to compete with other closely related *P. syringae* in order to colonize and infect *Populus*. Similarly, the oligopeptide mangotoxin, an NRP, acts as a more broad-spectrum antibiotic [58]. Ectoine protects the producing organism against osmotic and cold stress, both of which the bacteria may encounter in the phyllosphere and in the water cycle [59]. Thus, the biosynthetic potential of both *Populus* isolates reveals the pathogens are capable of effective survival and competition within the phyllosphere to colonize and subsequently infect the host.

### 3.5. Pathogenicity of P. syringae Isolates

**Figure 3:**
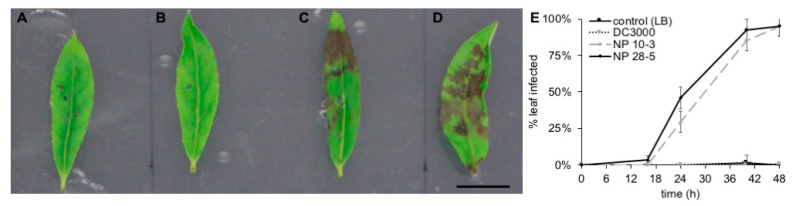
*Pseudomonas syringae* isolates from *P. trichocarpa* induce lesion formation on leaves. Leaves were cut from 3-month-old rooted *P. trichocarpa* BESC819, placed on agar infused with 1% glucose and 1× Hoagland’s basal salt mixture, and wounded 3× with a sterile pipette tip. Aliquots (10 µL, 8 × 10^5^ cells/mL) of (**A**) R2A media, (**B**) *P. syringae* pv. tomato DC3000, (**C**) *P. syringae* sp. NP10-3, or (**D**) *P. syringae* sp. NP28-5 were spotted onto each leaf wound and plates were incubated for 24 h at 25 °C. Scale bar, 10 mm. (**E**) Lesion development over 48 h on *P. trichocarpa* leaves. Error bars, standard deviation of three biological replicates with three leaves in each treatment group in each replicate.

After determining that the isolates were capable of inhibiting seed germination, we set out to investigate pathogenicity in poplar seedlings, as the pathogen is often found on leaf surfaces, stems, and fruit. First, leaf cuttings from 3-month-old rooted *P. trichocarpa* were wounded with a sterile syringe and challenged with bacterial isolates. The leaves treated with 10 µL aliquots of 8 × 10^5^ cells/mL NP10-3 or NP28-5 developed significant lesions within 24 h of treatment, indicating a spread of disease associated with the presence of the bacterial isolates (Figure 3A–E). Lesions were dark brown, spreading from the sites of application. In contrast, tomato pathovar *P. syringae* pv. tomato DC3000 (hereafter DC3000) did not produce lesions on leaves, even after extended treatment times of up to 7 days.

Additionally, 3-week-old rooted cuttings of *P. trichocarpa* planted in sterile soil developed lesions on the leaves when challenged with NP10-3 and NP28-5 on the underside of leaf surfaces, but to a much lesser extent when exposed to DC3000 (Figure S3A–D). Lesions on the leaves were observed within 24 h of treatment and spread across leaf surfaces over the course of 3 weeks. Small lesions were observed on DC3000-treated plants only after 2 weeks, indicating that this pathogen is much less suited to *P. trichocarpa* than NP10-3 and NP28-5. Above- and belowground masses, as well as chlorophyll content, did not differ significantly from control-treated plants (Table S5), which suggests that although the plants were responding to bacterial infection, they were still able to continue to dedicate resources to growth and development.

**Figure 4:**
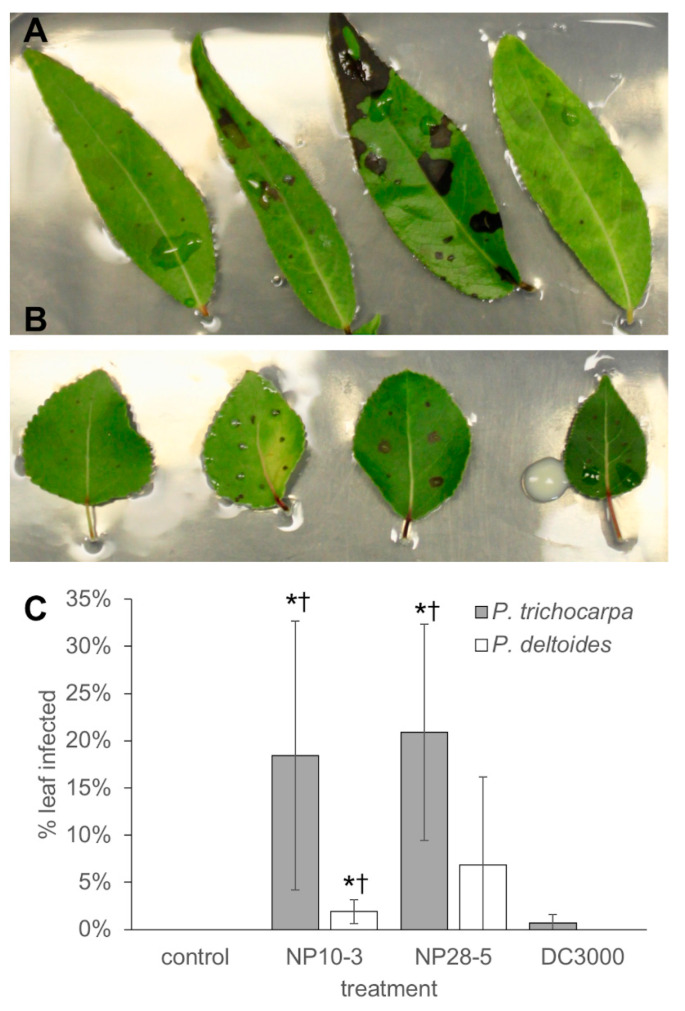
Comparison of *Populus* susceptibility to *P. syringae.* Surface-sterilized leaves were wounded six times with a sterile needle and 10 µL of bacteria (8 × 10^6^ cells/mL) was spotted on top of each wound. (**A**) *P. trichocarpa* Nisqually-1 leaves treated with (left to right) media control (R2A), NP10-3, NP28-5, and DC3000 for 48 h. (**B**) *P. deltoides* WV94 leaves treated with (left to right) media control, NP10-3, NP28-5, and DC3000 for 48 h. (**C**) Lesion development after 48 h. Error bars, standard deviation of four biological replicates. Grey, lesion development on *P. trichocarpa* leaves. White, lesion development on *P. deltoides* leaves. *, *p* < 0.05 compared to control; †, *p* < 0.05 compared to DC3000. Differences between NP10-3 and NP28-5 are not significant.

*P. syringae* pathovars are often limited in host range, although a previous study of 265 isolates from *P. tremuloides* and other woody plants revealed the majority of isolates triggered the HR in tomato (62%) and tobacco (79%) [30]. To this end, we surveyed the ability of NP10-3 and NP28-5 to elicit leaf lesion in a range of other plants, including two additional *Populus* species, tomato, and tobacco. NP10-3 and NP28-5 cause lesions on the leaves of other members of the poplar genus, *P. deltoides* and *P. tremuloides* (Table 4, Figure 4). Similar to DC3000, whose host range extends beyond tomato species into *Arabidopsis*, NP10-3 and NP28-5 rapidly result in *Arabidopsis* seedling death (Figure S3E–G). However, no lesion formation was observed on the leaves of other, non-*Populus* species, including *Phaseolus vulgaris* (bean), *Pisum sativum* (pea), *Nicotiana tabacum* (tobacco), and *Solanum lycopersicum* (tomato) (Table 4).

### 3.6. IAA and Motility Assays

*P. syringae* isolates are able to establish an infection in the host plant through a variety of mechanisms, including communication with the plant, attachment, and motility. To this end, we tested the ability of NP10-3 and NP28-5 to produce indole-3-acetic acid (IAA), a ubiquitous phytohormone which induces tissue differentiation, lateral root formation and root elongation, among other physiological effects [60]. At least three pathways for the production of IAA have been identified in bacteria, demonstrating its importance in plant-microbe interactions [61]. Compared to the beneficial *Populus* isolate *Pseudomonas* sp. GM30 [62,63] and the model strain DC3000, the pathogenic *P. syringae* produce significantly more IAA (Figure S1B). NP10-3 production of IAA is about twice that of NP28-5 under identical conditions. Both strains may rely heavily on IAA to manipulate *Populus* transcription and promotion of root production in order to establish an infection.

Motility and attachment through biofilm formation also give selective advantage to pathogenic bacteria and are known characteristics of *P. syringae* [64,65]. To confirm these phenotypes, we first identified motility genes in both organisms, which had nearly 100% conservation to the *P. syringae* pv. *syringae* B728a flagellar genes (B728a IMG Gene IDs 2508864182–2508864228; NP10-3 IMG Gene IDs 2758141872–2758141918; NP28-5 IMG Gene IDs 2757797404–2757797451). Next, we conducted motility assays and confirmed that both strains isolated from *Populus* produce a biofilm and are capable of motility, dispersing in LB 0.3% agar (Figure S4). Thus, the newly identified pathovars display many of the common phenotypes of *P. syringae.*

### 3.7. Metabolomics of Populus Trichocarpa in Response to P. syringae

In order to determine the plant host response to bacterial colonization, we first determined an appropriate time scale for observing leaf necrosis. As previously mentioned, leaf wounding using a sterilized syringe followed by application of NP10-3 and NP28-5 cultures resulted in observation of lesions on the leaves within 24–48 h, but control plants did not form lesions after wounding. We thus elected to collect metabolite samples from plants at 0 h, 24 h, and 48 h post-inoculation. Plants were wounded with a sterile syringe and treated with NP10-3 or NP28-5, while control plants were treated with sterile media. After the specified time points, leaves were harvested and analyzed for changes in metabolite profiles.

The effects of wounding leaves alone on plant metabolite response were more pronounced after 24 h than after 48 h. Thus, the effect on the plant from leaf wounding itself has only a minor contribution to changes in *Populus* leaf metabolites 48 h post-inoculation, so metabolite response to the pathogens was considered (Table S6). The plant defense response is highly induced when challenged with NP10-3 and NP28-5 compared to control and compared to initial metabolite levels (Table S6). This is especially evident in the accumulation of aromatics, including catechin (+4.02 FC for NP28-5 after 48 h), and particularly higher-order salicylates, including salicortin (+2.35 FC for NP10-3, +3.75 for NP28-5 after 48 h), 2,5-dihydroxybenzoic acid-5-O-glucosde (+5.04 FC for NP10-3, +2.77 for NP28-5 after 48 h), α-salicyloylsalicin (+2.24 FC for NP10-3, +3.55 FC for NP28-5 after 48 h), salicylic acid glucoside (+3.38 for NP28-5 after 48 h), salicyl-salicylic acid-2-O-glucoside (+2.40 for NP28-5 after 48 h), and trichocarpin (+2.22 for NP28-5 after 48 h), which are part of the constitutive defense response in *Populus* [48]. Accumulation remains about the same or slightly decreased for most metabolites between 24 and 48 h, indicating that at the earlier time point the plant is already sensing and responding to pathogen-associated cues and stressors. As seen in leaf infection models (Figure 3 and Figure 4), the plant response to NP28-5 is more pronounced than that induced by NP10-3, with more significantly accumulated metabolites (20 vs. 18) and higher fold change values in NP28-5 treated plants (Table S6). For example, digalactosylglycerol increased 3.92-fold after 48 h when NP28-5 was present, but only 2.57-fold in the presence of NP10-3. It is important to note that while catechol glucoside values are not statistically significant, in two plants within each dataset the metabolite was elevated in *Pseudomonas*-treated plants beyond the upregulated values observed in control plants. Overall, the *Pseudomonas* isolates quickly elicited the plant defense response as evidenced by the alteration of metabolite production.

## 4. Discussion

While *Populus* spp. have been extensively studied as a bioenergy crop, relatively few bacterial pathogens have thus far been described for use in model studies. Here, two strains of *Pseudomonas syringae* were isolated from *Populus* catkins in the field and characterized for host range, virulence genes, and host response. The full genome sequences of the isolates were used to positively identify both organisms as *Populus*-specific pathovars and inform potential host pathogenicity factors.

Importantly, NP10-3 and NP28-5 differ from a beneficial *Pseudomonas* strain isolated from the roots of *Populus* (*Pseudomonas* sp. GM17 [33]) in their ability to negatively affect the germination of *P. trichocarpa* seeds. Plant growth-promoting bacteria have been shown to increase the germination of wheat seeds in some cases [66]; although the beneficial *P. deltoides* isolate *Pseudomonas* sp. GM17 does not increase germination efficiency of *P. trichocarpa*. In contrast, NP10-3 and NP28-5 are pathogens that decrease germination efficiency.

An epiphytic pathogen [67], *P. syringae* was subsequently tested on both leaf cuttings and the leaves of rooted *Populus trichocarpa* seedlings. The treatment resulted in necrosis and the formation of lesions on the leaves of all seedlings treated with NP10-3 and NP28-5. Although genetic analysis shows a lack of the woody host and *Pseudomonas* species (WHOP) genes in NP28-5, the bacterium is still capable of infecting poplar seedlings, which confirms the previous observation that the WHOP genes are not a requirement of woody host pathogens [22]. However, infection studies on mature, woody plants may reveal differences in pathogenicity between the strains.

Interestingly, the effector protein hopBE1, present in both *Populus* isolates and found exclusively in woody host isolates [68], appears to be the product of horizontal gene transfer (HGT) due to its low GC content compared to the complete genomes of NP10-3 and NP28-5. Hop effectors not shared between NP10-3 and NP28-5 all have divergent GC content, indicative of HGT. The different effector protein content may contribute to the differences in virulence observed between the two isolates [69]; for example, HopAF1 from DC3000 has been shown to suppress plant immunity and is only present in the more virulent NP28-5 [70,71].

Both isolates were effective in inducing lesions on the leaves of other *Populus* species as well as disease in *P. trichocarpa* seedling stem wounds. *P. syringae* pathovars are generally plant-specific, having a narrow host range, yet many woody plant-specific isolates also elicit the HR in tobacco and tomato plants [30]. In contrast, NP10-3 and NP28-5 are limited in host specificity, with infection possible across all *Populus* species tested as well as in *Arabidopsis*, but no infection was observed in the other agricultural crops tested (Table 2). The native host, *P. trichocarpa*, was more susceptible than the related *P. deltoides*, which has a higher basal content of higher-order salicylates than *P. trichocarpa* and may explain its decreased susceptibility [48].

Differences in the TTEs, as well as expression of other virulence factors, likely contribute not just to the host range, but also the efficacy of the strain in establishing an infection in *Populus.* Whereas the suite of TTEs does not determine host range, a shared set of 18 conserved TTEs between NP10-3 and NP28-5 supports their similar host specificity. Bioinformatic analysis of the genomes revealed low numbers of TTEs, similar to other phylogroup II isolates, and four toxin-producing gene clusters, although only two toxins were produced in NP10-3 and NP28-5 monocultures. Despite having the WHOP cluster, NP10-3 pathogenicity on *P. trichocarpa* leaves was lower than that of NP28-5. While both isolates caused lesions on *P. trichocarpa* leaves and rooted cuttings, the genomic differences leading to variable pathogenicity remain to be fully elucidated.

Disruption of plant metabolic homeostasis is a well-understood phenomenon that occurs in response to stressors such as environmental changes, herbivory, and pathogens [72]. When an infection of *Populus* leaves occurs, the plant responds by inducing a stress response, as evidenced by the production of specific metabolites, including catechin, digalactosylglycerol and monogalactosylglycerol, salicortin and a number of other higher-order salicylates and closely related metabolites. Higher order salicylates, like salicortin, and their degradation products are common in poplar trees, and are important anti-inflammatory and antibiotic agents [73]. Relatedly, the kiwifruit salicylic acid-dependent defense response suppresses disease incidence and severity caused by *P. syringae* [73]. An increased production of higher-order salicylates within 48 h of treatment indicates that the infected host mounts an early defense response to the pathogens by further accumulating metabolites that are typically a major component of their constitutive defense network. Wounding alone without subsequent infection increased only isosalicin and 2,-5-dihydroxybenzoic acid-5-O-glucoside significantly (+2.73 FC and +2.20 FC, respectively), indicating that the induced stress response is due to bacterial infection rather than leaf wounding. Interestingly, the *P. syringae* isolate lacking the WHOP region, NP28-5, induced larger leaf lesions and greater changes in metabolite production, demonstrating that the cluster is not necessary for infection and that a more complex interplay of virulence factors and metabolic pathways are responsible for infection severity.

## Figures and Tables

**Table 1:** Bacterial strains reported for the first time in this study.

Species	Strain Name	Isolation Site	Host	Source	Accession
*Pseudomonas syringae*	NP10-3	ID, USA	*Populus trichocarpa*	G Newcombe	2757320439
*Pseudomonas syringae*	NP28-5	ID, USA	*Populus trichocarpa*	G Newcombe	2757320523

**Table 2:** General features of the *Pseudomonas syringae* NP10-3 and NP28-5 genomes.

Chromosome Features ^a^	NP10-3	NP28-5
genome size (bp)	6045676	5895985
plasmid (bp)	-	-
DNA coding sequence (%)	5358015 (88.63%)	5215021 (88.45%)
GC content (%)	59.24%	59.28%
total genes	5331	5116
protein coding genes	5202 (97.6%)	4985 (97.4%)
rRNA genes (5S rRNA, 16S rRNA, 23S rRNA)	9 (7, 1, 1)	9 (7, 1, 1)
tRNA	56	55
other RNA genes	64	67
total RNA genes	129	131
genes with assigned function	4338 (81.37%)	4213 (82.35%)
genes without assigned function	864 (16.21%)	772 (15.09%)
number of predicted enzymes	1337 (25.08%)	1320 (25.80%)
number of predicted effectors	21	20
biosynthetic clusters	19	17
genes in biosynthetic clusters	298 (5.59%)	301 (5.88%)
ANI ^b^, *P. syringae* pv. *syringae* B728a	98.79%	98.78%

^a^ Features for the genomes were identified from sequenced and annotated genomes on IMG/ER. ^b^ ANI, Average Nucleotide Identity.

**Table 3:** Genes located within the woody host and *Pseudomonas* species (WHOP) region identified in *P. syringae* isolates.

Function	Gene	Annotated Gene Product	NP10-3 Gene ID	% ID ^a^	NP28-5 Gene ID
Catechol catabolism	*catA*	Catechol 1,2-dioxygenase	2758144716	82	ND ^b^
	*catC*	Muconolactone isomerase	2758144717	82	ND
	*catB*	Muconate cycloisomerase	2758144718	89	ND
Anthranilate catabolism	*antC*	Anthranilate dioxygenase reductase component	2758144712	39	ND
	*antB*	Anthranilate dioxygenase beta subunit	2758144713	36	ND
	*antA*	Anthranilate dioxygenase alpha subunit	2758144714	47	ND
	*antR*	*antABC* regulatory protein	2758144715	25	ND
Indigo-producing oxygenase	*ipoC*	Involved in *meta* pathway of phenol degradation	ND	NA ^c^	ND
	*ipoB*	Nitrilotriacetate monooxygenase component B	ND	NA	ND
	*ipoA*	Putative oxygenase subunit	ND	NA	ND
Not determined		Aerotaxis receptor	ND	NA	ND
	*dhoB*	Short chain alcohol dehydrogenase	ND	NA	ND
	*dhoA*	Dienelactone hydrolase	ND	NA	ND
	*benR*	Positive regulator of the *benABCD* operon	ND	NA	ND

^a^ %ID corresponding to the gene identified in *P. savastanoi* pv. *savastanoi* NCPPB 3335. ^b^ ND. No similar gene determined. ^c^ NA. Not applicable.

**Table 4:** Scope of pathogenicity of *Populus trichocarpa* isolates NP10-3 and NP28-5. *Pseudomonas syringae* pv. tomato DC3000 was used as a comparison.

Host	DC3000	NP10-3	NP28-5
*P. trichocarpa* BESC819	N	Y	Y
*P. trichocarpa* Nisqually-1	N	Y	Y
*P. deltoides* WV94	N	Y	Y
*Arabidopsis thaliana* Col-1	Y	Y	Y
Avocado (*Persea americana* pv. Hass)	N	N	N
Bean (*Phaseolus vulgaris* pv. Tenderette)	N	N	N
Pea (*Pisum sativum* pv. Macrocarpon)	N	N	N
Spinach (*Spinacia oleracea* pv. Bloomsdale Longstanding)	N	N	N
Tobacco (*Nicotiana tabacum* pv. Little Crittenden)	N	N	N
Tomato (*Solanum lycopersicum* pv. Brandywine)	Y	N	N
Wheat (*Triticum aestivum* pv. Winter Wheat)	N	N	N

## Data Availability

The genomes for NP10-3 and NP28-5 have been deposited in the JGI-IMG database (https://img.jgi.doe.gov/) under ID codes 2757320439 and 2757320523, and the GenBank database under accessions QKUC00000000, QICP00000000, respectively.

## Supplementary Materials

The following are available online at https://www.mdpi.com/2076-2607/8/8/1137/s1, **Figure S1.** Culture conditions of *Pseudomonas syringae* isolates, **Figure S2.** Toxins detected in *P. syringae* culture supernatants and the biosynthetic gene clusters (BGCs) found in *P. syringae* genomes, **Figure S3.** *Pseudomonas syringae* isolates cause leaf lesions in 3-week-old *P. trichocarpa* seedlings and kill 3-day-old germinated *Arabidopsis thaliana* seedlings, **Figure S4.** Motility of *Pseudomonas syringae* strains on LB 0.3% agar after 24 h, **Table S1.** Gene IDs and % identity to the *P. syringae* B728a sequence for *P. syringae* phylogroup II-specific genes, **Table S2.** Effector protein loci and IMG gene IDs for *Pseudomonas* strains, **Table S3.** GC content of effector proteins compared to the GC content of the genomes of *P. syringae* NP10-3 and *P. syringae* NP28-5, **Table S4.** Antibiotic susceptibility of *P. syringae* poplar isolates compared to *P. syringae* pv. *tomato* DC3000, **Table S5.** Physiological effects on *Populus trichocarpa* rooted seedlings after 15 d exposure to *Pseudomonas syringae* isolates, **Table S6.** Metabolite profile of control and *Pseudomonas*-infected *Populus trichocarpa* rooted plants.

## References

[1] Xin X.F., Kvitko B., He S.Y. (2018). Pseudomonas syringae: What it takes to be a pathogen. Nat. Rev. Microbiol..

[2] Hwang M.S.H., Morgan R.L., Sarkar S.F., Wang P.W., Guttman D.S. (2005). Phylogenetic characterization of virulence and resistance phenotypes of Pseudomonas syringae. Appl. Environ. Microbiol..

[3] Hirano S.S., Upper C.D. (2000). Bacteria in the leaf ecosystem with emphasis on Pseudomonas syringae—A pathogen, ice nucleus, and epiphyte. Microbiol. Mol. Biol. Rev..

[4] Block A., Li G., Fu Z.Q., Alfano J.R. (2008). Phytopathogen type III effector weaponry and their plant targets. Curr. Opin. Plant Biol..

[5] Galán J.E., Collmer A. (1999). Type III secretion machines: Bacterial devices for protein delivery into host cells. Science.

[6] Collmer A., Badel J.L., Charkowski A.O., Deng W.L., Fouts D.E., Ramos A.R., Rehm A.H., Anderson D.M., Schneewind O., van Dijk K. (2000). Pseudomonas syringae Hrp type III secretion system and effector proteins. Proc. Natl. Acad. Sci. USA.

[7] Morel J.B., Dangl J.L. (1997). The hypersensitive response and the induction of cell death in plants. Cell Death Differ..

[8] Nurnberger T., Nennstiel D., Jabs T., Sacks W.R., Hahlbrock K., Scheel D. (1994). High affinity binding of a fungal oligopeptide elicitor to parsley plasma membranes triggers multiple defense responses. Cell.

[9] Buell C.R., Joardar V., Lindeberg M., Selengut J., Paulsen I.T., Gwinn M.L., Dodson R.J., Deboy R.T., Durkin A.S., Kolonay J.F. (2003). The complete genome sequence of the Arabidopsis and tomato pathogen Pseudomonas syringae pv. tomato DC3000. Proc. Natl. Acad. Sci. USA.

[10] Ward J.L., Forcat S., Beckmann M., Bennett M., Miller S.J., Baker J.M., Hawkins N.D., Vermeer C.P., Lu C., Lin W. (2010). The metabolic transition during disease following infection of Arabidopsis thaliana by Pseudomonas syringae pv. tomato. Plant J..

[11] Rico A., McCraw S.L., Preston G.M. (2011). The metabolic interface between Pseudomonas syringae and plant cells. Curr. Opin. Microbiol..

[12] Lindeberg M., Cunnac S., Collmer A. (2009). The evolution of Pseudomonas syringae host specificity and type III effector repertoires. Mol. Plant Pathol..

[13] Chisholm S.T., Coaker G., Day B., Staskawicz B.J. (2006). Host-microbe interactions: Shaping the evolution of the plant immune response. Cell.

[14] Helmann T.C., Deutschbauer A.M., Lindow S.E. (2019). Genome-wide identification of Pseudomonas syringae genes required for fitness during colonization of the leaf surface and apoplast. Proc. Natl. Acad. Sci. USA.

[15] Rohmer L., Guttman D.S., Dangl J.L. (2004). Diverse Evolutionary Mechanisms Shape the Type III Effector Virulence Factor Repertoire in the Plant Pathogen Pseudomonas syringae. Genetics.

[16] Bender C.L., Alarcón-Chaidez F., Gross D.C. (1999). Pseudomonas syringae phytotoxins: Mode of action, regulation, and biosynthesis by peptide and polyketide synthetases. Microbiol. Mol. Biol. Rev..

[17] Block A., Schmelz E., Jones J.B., Klee H.R. (2005). Coronatine and salicylic acid: The battle between Arabidopsis and Pseudomonas for phytohormone control. Mol. Plant Pathol..

[18] Uppalapati S.R., Ishiga Y., Wangdi T., Urbanczyk-Wochniak E., Ishiga T., Mysore K.S., Bender C.L. (2008). Pathogenicity of Pseudomonas syringae pv. tomato on tomato seedlings: Phenotypic and gene expression analyses of the virulence function of coronatine. Mol. Plant. Microbe Interact..

[19] Ramos C., Matas I.M., Bardaji L., Aragon I.M., Murillo J. (2012). Pseudomonas savastanoi pv. savastanoi: Some like it knot. Mol. Plant Pathol..

[20] Green S., Studholme D.J., Laue B.E., Dorati F., Lovell H., Arnold D., Cottrell J.E., Bridgett S., Blaxter M., Huitema E. (2010). Comparative genome analysis provides insights into the evolution and adaptation of Pseudomonas syringae pv. aesculi on Aesculus hippocastanum. PLoS ONE.

[21] Sarkar S.F., Gordon J.S., Martin G.B., Guttman D.S. (2006). Comparative genomics of host-specific virulence in Pseudomonas syringae. Genetics.

[22] Caballo-Ponce E., van Dillewijn P., Wittich R.M., Ramos C. (2017). WHOP, a genomic region associated with woody hosts in the Pseudomonas syringae complex contributes to the virulence and fitness of Pseudomonas savastanoi pv. savastanoi in olive plants. Mol. Plant Microbe Interact..

[23] Otto M., Hammerbacher A., Petersen Y., Pierneef R., Coutinho T.A. (2018). Phenolic compound degradation by Pseudomonas syringae phylogroup 2 strains. J. Plant Pathol..

[24] Lamichhane J.R., Varvaro L., Parisi L., Audergon J.M., Morris C.E., Sparks D.L. (2014). Chapter Four. Disease and Frost Damage of Woody Plants Caused by Pseudomonas syringae: Seeing the Forest for the Trees. Advances in Agronomy.

[25] Hulin M.T., Armitage A.D., Vicente J.G., Holub E.B., Baxtger L., Bates H.J., Mansfield J.W., Jackson R.W., Harrison R.J. (2018). Comparative genomics of Pseudomonas syringae reveals convergent gene gain and loss associated with specialization onto cherry (Prunus avium). New Phytol..

[26] Tuskan G.A., DiFazio S., Jansson S., Bohlmann J., Grigoriev I., Hellsten U., Putnam N., Ralph S., Rombauts S., Salamov A. (2006). The genome of black cottonwood, Populus trichocarpa. Science.

[27] Ramstedt M., Årström B., Fircks H.A. (1994). Dieback of poplar and willow caused by Pseudomonas syringae in combination with freezing stress. Eur. J. Forest Pathol..

[28] Haworth R.H., Spiers A.G. (1988). Characterisation of bacteria from poplars and willows exhibiting leaf spotting and stem cankering in New Zealand. Eur. J. Forest Pathol..

[29] Morris C.E., Sands D.C., Vinatzer B.A., Glaux C., Guilbaud C., Buffière A., Yan S., Dominguez H., Thompson B.M. (2008). The life history of the plant pathogen Pseudomonas syringae is linked to the water cycle. ISME J..

[30] Canfield M.L., Baca S., Moore L.W. (1986). Isolation of Pseudomonas syringae from 40 cultivars of diseased woody plants with tip dieback in Pacific Northwest nurseries. Plant Dis..

[31] Abraham P.E., Garcia B.J., Gunter L.E., Jawdy S.S., Engle N.L., Yang X., Jacobson D.A., Hettich R.L., Tuskan G.A., Tschapliknski T.J. (2018). Quantitative proteome profile of water deficit stress responses in eastern cottonwood (Populus deltoides) leaves. PLoS ONE.

[32] Timm C.M., Pelletier D.A., Jawdy S.S., Gunter L.E., Henning J.A., Engle N.L., Aufrecht J., Gee E., Nookaew I., Yang Z. (2016). Two poplar-associated bacterial isolates induce additive favorable responses in a constructed plant-microbiome system. Front. Plant Sci..

[33] Brown S.D., Utturkar S.M., Klingeman D.M., Johnson C.M., Martin S.L., Land M.L., Lu T.Y., Schadt C.W., Doktycz M.J., Pelletier D.A. (2012). Twenty-one genome sequences from Pseudomonas species and 19 genome sequences from diverse bacteria isolated from the rhizosphere and endosphere of Populus deltoides. J. Bacteriol..

[34] Markowitz V.M., Mavromatis K., Ivanova N.N., Chen I.M., Chu K., Kyrpides N.C. (2009). IMG ER: A system for microbial genome annotation expert review and curation. Bioinformatics.

[35] Markowitz V.M., Chen I., Min A., Palaniappan K., Chu K., Szeto E., Grechkin Y., Ratner A., Jacob B., Huang J. (2012). IMG: The integrated microbial genomes database and comparative analysis system. Nucleic Acids Res..

[36] Lawrence T.J., Kauffman K.T., Amrine K.C.H., Carper D.L., Lee R.S., Becich P.J., Canales C.J., Ardell D.H. (2015). FAST: FAST Analysis of Sequences Toolbox. Front. Genet..

[37] Kalyaanamoorthy S., Minh B.Q., Wong T.K.F., von Haeseler A., Jermiin L.S. (2017). ModelFinder: Fast model selection for accurate phylogenetic estimates. Nat. Methods.

[38] Le S.Q., Gascuel O. (2008). An Improved General Amino Acid Replacement Matrix. Mol. Biol. Evol..

[39] Yang Z. (1995). A space-time process model for the evolution of DNA sequences. Genetics.

[40] Yang Z. (1994). Maximum likelihood phylogenetic estimation from DNA sequences with variable rates over sites: Approximate methods. J. Mol. Evol..

[41] Soubrier J., Steel M., Lee M.S.Y., Der Sarkissian C., Guindon S., Ho S.Y.W., Cooper A. (2012). The Influence of Rate Heterogeneity among Sites on the Time Dependence of Molecular Rates. Mol. Biol. Evol..

[42] Nguyen L.-T., Schmidt H.A., von Haeseler A., Minh B.Q. (2014). IQ-TREE: A Fast and Effective Stochastic Algorithm for Estimating Maximum-Likelihood Phylogenies. Mol. Biol. Evol..

[43] Minh B.Q., Nguyen M.A.T., von Haeseler A. (2013). Ultrafast Approximation for Phylogenetic Bootstrap. Mol. Biol. Evol..

[44] Hoang D.T., Chernomor O., von Haeseler A., Minh B.Q., Vinh L.S. (2017). UFBoot2: Improving the Ultrafast Bootstrap Approximation. Mol. Biol. Evol..

[45] Wolfe A.J., Berg H.C. (1989). Migration of bacteria in semisolid agar. Proc. Natl. Acad. Sci. USA.

[46] O’Toole G.A. (2011). Microtiter dish biofilm formation assay. J. Vis. Exp..

[47] Tschaplinski T.J., Plett J.M., Engle N.L., Deveau A., Cushman K.C., Martin M.Z., Doktycz M.J., Tuskan G.A., Brun A., Kohler A. (2014). Populus trichocarpa and Populus deltoides exhibit different metabolomic responses to colonization by the symbiotic fungus Laccaria bicolor. Mol. Plant Microbe Interact..

[48] Tschaplinski T.J., Standaert R.F., Engle N.L., Martin M.Z., Sangha A.K., Parks J.M., Smith J.C., Samuel R., Jiang N., Pu Y. (2012). Down-regulation of the caffeic acid O-methyltransferase gene in switchgrass reveals a novel monolignol analog. Biotechnol. Biofuels.

[49] Labbé J.L., Weston D.J., Dunkirk N., Pelletier D.A., Tuskan G.A. (2014). Newly identified helper bacteria stimulate ectomycorrhizal formation in Populus. Front. Plant Sci..

[50] Goris J., Konstantinidis K.T., Klappenbach J.A., Coenye T., Vandamme P., Tiedje J.M. (2007). DNA-DNA hybridization values and their relationship to whole-genome sequence similarities. Int. J. Syst. Evol. Microbiol..

[51] Dillon M.M., Thakur S., Almeida R.N.D., Wang P.W., Weir B.S., Guttman D.S. (2019). Recombination of ecologically and evolutionarily significant loci maintains genetic cohesion in the Pseudomonas syringae species complex. Genome Biol..

[52] Baltrus D.A., Nishimura M.R., ROmanchuk A., Chang J.H., Mukhtar M.S., Cherkis K., Roach J., Grant S.R., Jones C.D., Dangl J.L. (2011). Dynamic evolution of pathogenicity revealed by sequencing and comparative genomics of 19 Pseudomonas syringae isolates. PLoS Pathog..

[53] Schellenberg B., Ramel C., Dudler R. (2010). Pseudomonas syringae virulence factor syringolin A counteracts stomatal immunity by proteasome inhibition. Mol. Plant Microbe Interact..

[54] Hutchison M.L., Tester M.A., Gross D.C. (1995). Role of biosurfactant and ion channel-forming activities of syringomycin in transmembrane ion flux: A model for the mechanism of action in the plant-pathogen interaction. Mol. Plant Microbe Interact..

[55] Quinones B., Dulla G., Lindow S.E. (2005). Quorum sensing regulates exopolysaccharide production, motility, and virulence in Pseudomonas syringae. Mol. Plant Microbe Interact..

[56] Braun S.D., Voksch B., Nuske J., Spiteller D. (2008). 3-Methylarginine from Pseudomonas syringae pv. syringae 22d/93 suppresses the bacterial blight caused by its close relative Pseudomonas syringae pv. glycinea. ChemBioChem.

[57] Arrebola E., Cazorla F.M., Durán V.E., Rivera E., Olea F., Codina J.C., Pérez-García A., de Vicente A. (2003). Mangotoxin: A novel antimetabolite toxin produced by Pseudomonas syringae inhibiting ornithine/arginine biosynthesis. Physiol. Mol. Plant Pathol..

[58] Kuhlmann A.U., Hoffmann T., Bursy J., Jebbar M., Bremer E. (2011). Ectoine and hydroxyectoine as protectants against osmotic and cold stress: Uptake through the SigB-controlled betaine-choline- carnitine transporter-type carrier EctT from Virgibacillus pantothenticus. J. Bacteriol..

[59] Duca D., Lorv J., Patten C.L., Rose D., Glick B.R. (2014). Indole-3-acetic acid in plant-microbe interactions. Antonie Van Leeuwenhoek.

[60] Spaepen S., Vanderleyden J. (2011). Auxin and plant-microbe interactions. Cold Spring Harb. Perspect. Biol..

[61] Weston D.J., Pelletier D.A., Morrell-Falvey J.L., Tschaplinski T.J., Jawdy S.S., Lu T.Y., Allen S.M., Melton S.J., Martin M.Z., Shadt C.W. (2012). Pseudomonas fluorescens induces strain-dependent and strain-independent host plant responses in defense networks, primary metabolism, photosynthesis, and fitness. Mol. Plant Microbe Interact..

[62] Timm C.M., Campbell A.G., Utturkar S.M., Jun S.-R., Parales R.E., Tan W.A., Robeson M.S., Lu T.S., Jawdy S.S., Brown S.D. (2015). Metabolic functions of Pseudomonas fluorescens strains from Populus deltoides depend on rhizosphere or endosphere isolation compartment. Front. Microbiol..

[63] Ghods S., Sims I.M., Moradali M.F., Rehm B.H. (2015). Bactericidal compounds controlling growth of the plant pathogen Pseudomonas syringae pv. actinidiae, which forms biofilms composed of a novel exopolysaccharide. Appl. Environ. Microbiol..

[64] Danhorn T., Fuqua C. (2007). Biofilm formation by plant-associated bacteria. Annu. Rev. Microbiol..

[65] Delshadi S., Ebrahimi M., Shirmohammadi E. (2017). Influence of plant-growth-promoting bacteria on germination, growth and nutrients’ uptake of Onobrychis sativa L. under drought stress. J. Plant Interact..

[66] Arnold D.L., Preston G.M. (2019). Pseudomonas syringae: Enterprising epiphyte and stealthy parasite. Microbiology.

[67] Nowell R.W., Laue B.E., Sharp P.M., Green S. (2016). Comparative genomics reveals genes significantly associated with woody hosts in the plant pathogen Pseudomonas syringae. Mol. Plant Pathol..

[68] Macho A.P., Zumaquero A., Gonzalez-Plaza J.J., Ortiz-Martín I., Rufián J.S., Beuzón C.R. (2012). Genetic analysis of the individual contribution to virulence of the type III effector inventory of Pseudomonas syringae pv. phaseolicola. PLoS ONE.

[69] Washington E.J., Mukhtar M.S., Finkel O.M., Wan L., Banfield M.J., Kieber J.J., Dangl J.L. (2016). Pseudomonas syringae type III effector HopAF1 suppresses plant immunity by targeting methionine recycling to block ethylene induction. Proc. Natl. Acad. Sci. USA.

[70] Li X., Lin H., Zhang W., Zou Y., Zhang J., Tang X., Zhou J.-M. (2005). Flagellin induces innate immunity in nonhost interactions that is suppressed by Pseudomonas syringae effectors. Proc. Natl. Acad. Sci. USA.

[71] Shulaev V., Cortes D., Miller G., Mittler R. (2008). Metabolomics for plant stress response. Physiol. Plant.

[72] Chen F., Liu C.-J., Tschaplinski T.J., Zhao N. (2009). Genomics of secondary metabolism in Populus: Interactions with biotic and abiotic environments. Crit. Rev. Plant Sci..

[73] Cellini A., Fiorentini L., Buriani G., Yu J., Donati I., Cornish D.A., Novak B., Costa G., Vanneste J.L., Spinelli F. (2014). Elicitors of the salicylic acid pathway reduce incidence of bacterial canker of kiwifruit caused by Pseudomonas syringae pv. actinidae. Ann. Appl. Biol..

