# Supplementary material for: Isolation, Characterization, and Pathogenicity of Two Pseudomonas syringae Pathovars from Populus trichocarpa Seeds
Source: Microorganisms. 2020 Jul 28;8(8):1137. doi: 10.3390/microorganisms8081137 (PMC7465253; doi:10.3390/microorganisms8081137)
Supplement: Supplementary file 1 [file microorganisms-08-01137-s001.pdf]

Supplemental Information
for
Isolation, characterization, and pathogenicity of two *Pseudomonas syringae* pathovars from *Populus trichocarpa* seeds

Patricia M.B. Saint-Vincent, Mary Ridout, Nancy L. Engle, Meredith L. Yeary, Timothy J. Tschaplinski, George Newcombe, and Dale A. Pelletier

Supplemental Figures.

Figure S1. Culture conditions of *Pseudomonas syringae* isolates. (A) Degradation analysis of catechol and anthranilate by *Pseudomonas syringae* NP10-3, NP28-5, and tomato pathovar DC3000. (B) Production of indole-3-acetic acid (IAA) in *Pseudomonas syringae* isolates and beneficial endophyte *Pseudomonas fluorescens* GM30. Error bars, standard deviation of three biological replicates, each measured in triplicate.

A

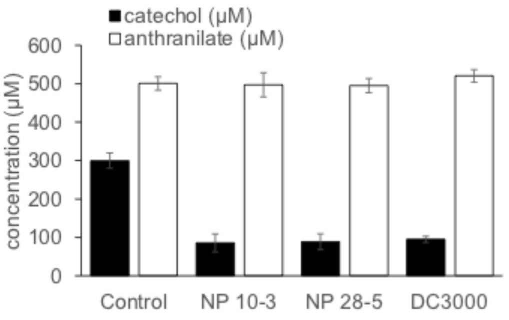

B

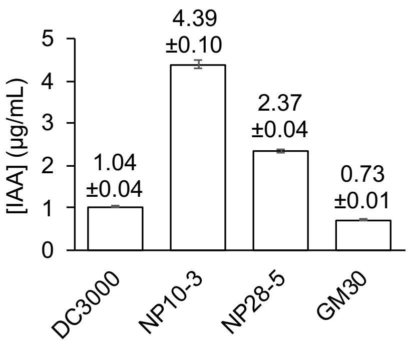

Figure S2. Toxins detected in *P. syringae* culture supernatants and the biosynthetic gene clusters (BGCs) found in *P. syringae* genomes. (A) Base peak total ion chromatogram (TIC) of *P. syringae* NP10-3. (B) Extracted ion chromatogram (EIC) of coronatine, *m/z* 319.1784, in *P. syringae* NP10-3. (C) EIC of syringolin, *m/z* 493.2900, in *P. syringae* NP10-3. (D) Base peak total ion chromatogram (TIC) of *P. syringae* NP28-5. (E) Extracted ion chromatogram (EIC) of coronatine, *m/z* 319.1784, in *P. syringae* NP28-5. (F) EIC of syringolin, *m/z* 493.2900, in *P. syringae* NP28-5. (G) BGCs of NP10-3 and NP28-5, predicted with antiSMASH 3.0. Clusters with >90% similarity to a known cluster are identified based on biosynthetic class.

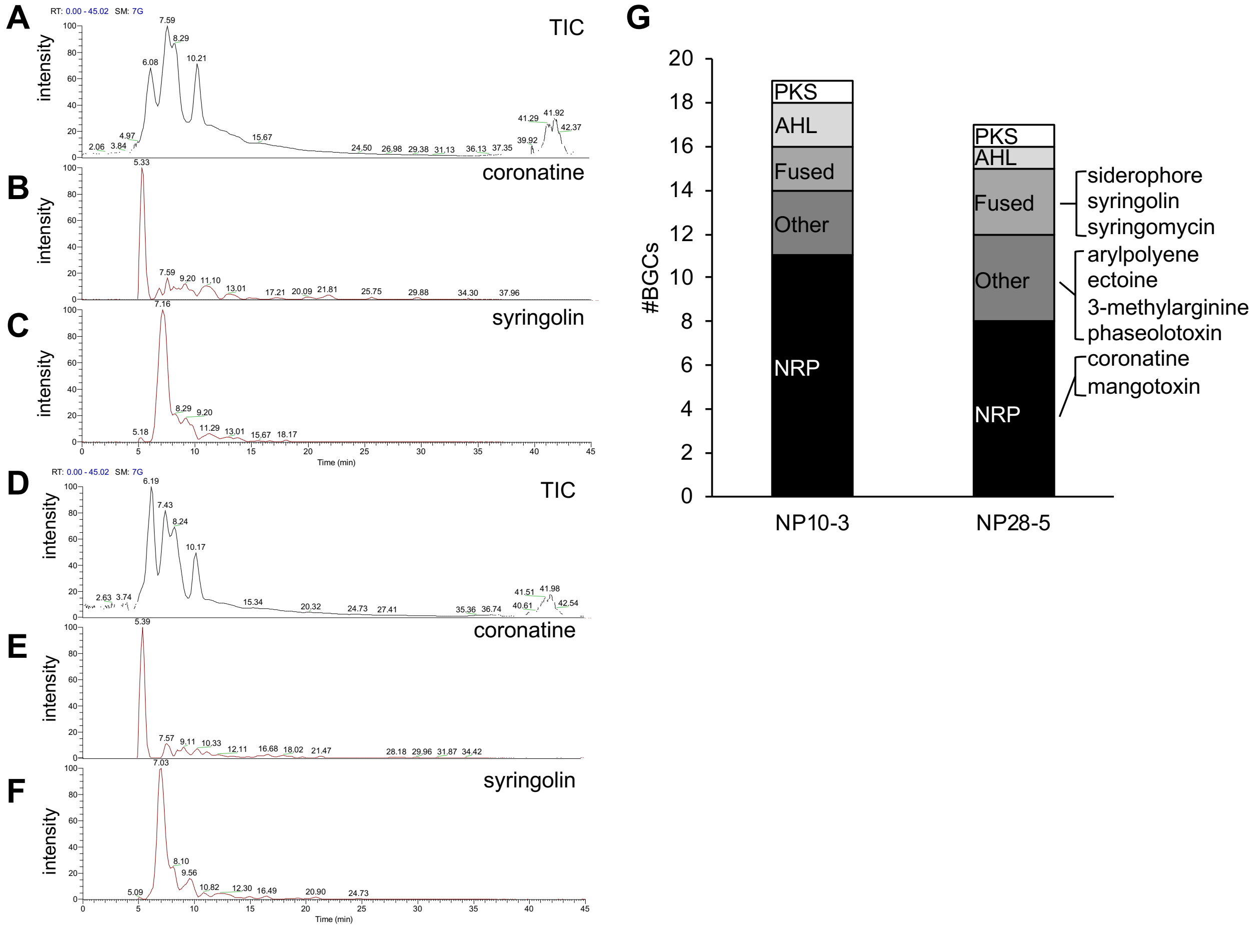

Figure S3. *Pseudomonas syringae* isolates cause leaf lesions in 3-week-old *P. trichocarpa* seedlings and kill 3-day-old germinated *Arabidopsis thaliana* seedlings. (A) Control-treated *P. trichocarpa* after 3 weeks. (B) *P. trichocarpa* inoculated with *P. syringae* pv. tomato DC3000. (C) *P. trichocarpa* inoculated with *P. syringae* NP10-3. (D) *P. trichocarpa* inoculated with *P. syringae* NP28-5. (E) Control-treated *Arabidopsis* after 2 weeks. (F) *Arabidopsis* inoculated with *P. syringae* NP10-3. (G) *Arabidopsis* inoculated with *P. syringae* NP28-5.

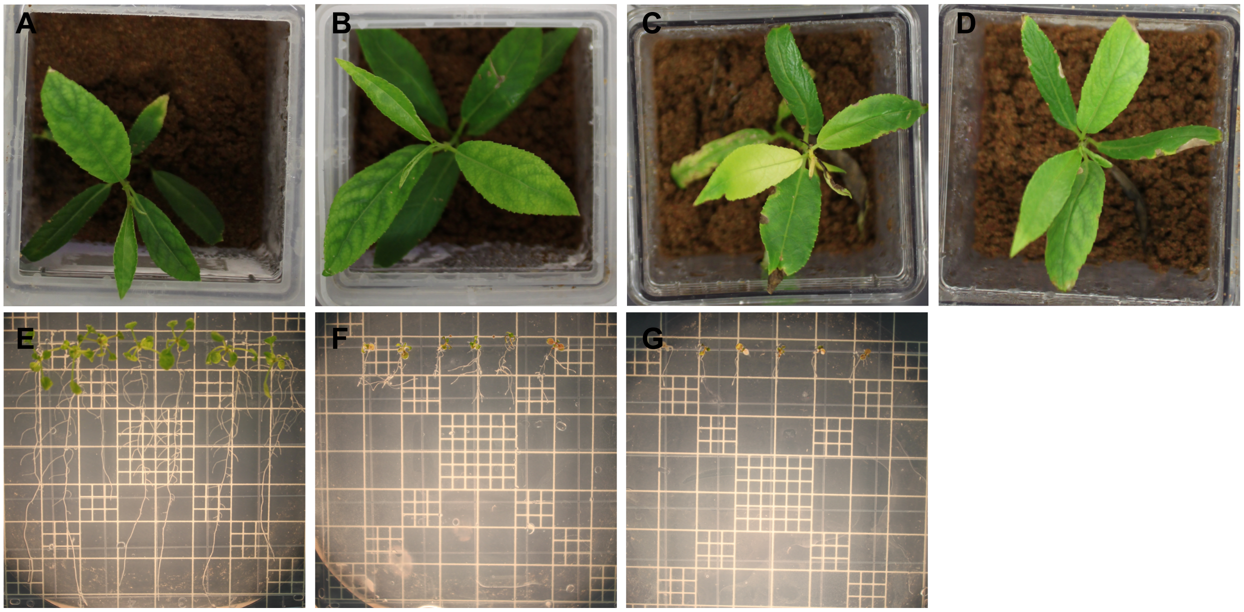

Figure S4. Motility of *Pseudomonas syringae* strains on LB 0.3% agar after 24 h. (A) *P. syringae* pv. *tomato* DC3000. (B) *P. syringae* sp. NP10-3. (C) *P. syringae* sp. NP28-5.

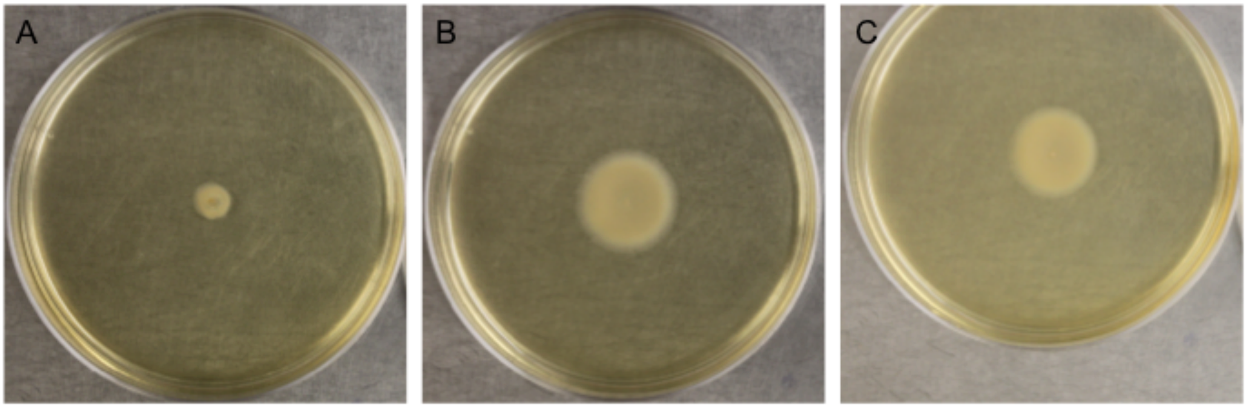

Supplemental Tables.

Table S1. Gene IDs and % identity to the *P. syringae* B728a sequence for *P. syringae* phylogroup II-specific genes.

**B728a Gene ID**	**Gene Name**	**AA**	**NP10-3 Gene ID**	**% ID**	**NP28-5 Gene ID**	**%ID**
2508861406	DNA-binding transcriptional regulator, LysR family	306	2758144526	99	2757798338	98
2508861408	predicted membrane protein, DUF421	147	2758144524	100	2757798340	100
2508861409	hypothetical protein	152	2758144523	99	2757798341	99
2508862882	sugar phosphate permease	440	2758144016	99	2757795041	99
2508862886	efflux transporter, outer membrane factor lipoprotein, NodT family	490	2758144021	99	2757795046	99
2508862989	DUF1810	141	2758144904	97	2757795154	97
2508863051	uncharacterized conserved protein YegJ	408	2758141542	100	-	-
2508863079	hypothetical protein	80	2758141514	100	2757795849	100
2508863081	hypothetical protein	143	2758141512	100	2757795847	100
2508863082	DUF72	244	2758141511	99	2757795846	99
2508863085	hypothetical protein	78	2758141508	100	2757795843	100
2508863302	AAA domain-containing protein	1945	2758141285	99	2757795616	99
2508863396	uncharacterized iron-regulated membrane protein	464	2758143825	99	2757798743	99
2508863651	GntR transcriptional regulator	239	2758142249	100	2757795301	100
2508863652	ADP-ribosylglycohydrolase	337	2758142250	100	2757795302	100
2508863654	ribokinase family protein	308	2758142252	99	2757795304	99
2508864240	methylase involved in ubiquinone/menaquinone biosynthesis	303	2758141860	99	2757787392	99
2508864241	hypothetical protein	269	2758141859	99	2757797391	100
2508864540	predicted dithiol-disulfide isomerase, DsbA family	210	2758144162	100	2757798627	100
2508865031	response regulator, CheY-like receiver domain and HTH DNA-binding domain	330	2758140915	99	2757798439	99
2508865261	ExbB outer membrane transport energization protein	242	2758141783	100	2757798838	100

Table S2. Effector protein loci and IMG gene IDs for *Pseudomonas* strains. Effectors from *Pseudomonas* sp. B728, B301D, and H191 are reported in Collmer et al.(1) and Baltrus et al(2).

Effector	B728 locus	B301D locus	HS191 locus	NP10-3 locus	% ID[1]	NP28-5 locus	% ID[1]
**avrA1**	-	-	-	-		-	
**avrB1**	-	-	-	-		-	
**avrB2**	-	-	-	-		-	
**avrB3**	Psyr_1219	-	-	-		-	
**avrB4**	-	-	-	-		-	
**avrD1**	-	-	-	-		-	
**avrE1**	Psyr_1188	PsyrB_06260	PsyrH_20365	2758140350	98	2757798191	97
**avrPto1**	Psyr_4919	-	-	-		-	
**avrPto2**	-	-	-	-		-	
**avrPto3**	-	-	-	-		-	
**avrPto4**	-	-	-	-		-	
**avrPto5**	-	-	-	-		-	
**avrRpm1**	Psyr_0738	-	-	-		-	
**avrRpm2**	-	-	-	-		-	
**avrRps4**	-	-	-	-		-	
**avrRpt2**	-	-	-	-		-	
**hopA1**	-	-	-	-		-	
**hopA2**	-	PsyrB_06425	PsyrH_20205	2758140318[3]	100	-	
**hopB1**	-	-	-	-		-	
**hopC1**	-	-	PsyrH_20070	-		-	
**hopD1**	-	-	-	-		-	
**hopE1**	-	-	-	-		-	
**hopF1**	-	-	-	-		-	
**hopF2**	-	-	-	-		-	
**hopF3**	-	-	-	-		-	
**hopG1**	-	-	-	-		-	
**hopH1**	Psyr_1889	PsyrB_09320	PsyrH_20075	2758143460	93	2757794720	93
**hopH2**	-	-	-	-		-	
**hopH3**	-	-	-	-		-	
**hopI1**	Psyr_4326	PsyrB_22470	PsyrH_04610	2758141612	98	2757799125	98
**hopJ1**	Psyr_1017	PsyrB_05270	PsyrH_21240	2758143360	100	2757797731	100
**hopK1**	-	-	-	-		-	
**hopM1**	Psyr_1186	PsyrB_06250	PsyrH_20375	2758140352	94	2757798193	93
**hopN1**	-	-	-	-		-	
**hopO1**	-	-	-	-		-	
**hopQ1**	-	-	-	-		-	
**hopR1**	-	-	PsyrH_12260	-		-	
**hopS1**	-	-	-	-		-	
**hopS2**	-	-	-	-		-	
**hopT1**	-	-	-	-		-	
**hopT2**	-	-	-	-		-	
**hopU1**	-	-	-	-		-	
**hopV1**	-	-	-	-		-	
**hopW1**	-	-	PsyrH_12235	2758140918[4]	49	2757798436[4]	48
**hopX1**	Psyr_1220	-	-	-		-	
**hopX2**	-	-	-	-		-	
**hopZ1**	-	-	PsyrH_20105	-		-	
**hopZ2**	-	-	-	-		-	
**hopZ3**	Psyr_1224	-	-	-		-	
**hopZ4**	-	-	-	-		-	
**hopZ5**	-	-	-	-		-	
**hopAA1**	Psyr_1183	PsyrB_06235	PsyrH_20390	2758140355	98	2757798196	98
**hopAB1**	Psyr_4659	-	-	-		-	
**hopAB2**	-	-	-	-		-	
**hopAB3**	-	-	-	-		-	

**hopAC1**	Psyr_0527	PsyrB_02970	PsyrH_02665	2758141118	99	2757796037	99
**hopAD1**	-	-	-	-		-	
**hopAE1**	Psyr_4269	PsyrB_22195	PsyrH_04865	2758140918	99	2757798436	99
**hopAF1**	Psyr_3813	-	PsyrH_07325	-		2757798647	97
**hopAG1**	Psyr_0778	PsyrB_04160	PsyrH_22420	2758144515	99	2757798349	99
**hopAH1**	Psyr_0779	PsyrB_04165	PsyrH_22410	2758144514	100	2757798350	99
**hopAH2**	Psyr_3123	PsyrB_16095	PsyrH_10955	2758144517	99	2757795508	98
**hopAI1**	Psyr_0785	PsyrB_04195	PsyrH_22380	2758144508	99	2757798356	99
**hopAJ2**	Psyr_4357	PsyrB_22625	PsyrH_04450	2758141643	99	2757799077	99
**hopAK1**	Psyr_3839	PsyrB_19950	PsyrH_07200	2758144763	99	2757798675	99
**hopAL1**	-	PsyrB_08740	-	-		2757794607[3]	99
**hopAM1**	-	-	-	-		-	
**hopAN1**	Psyr_0465	PsyrB_02660	PsyrH_02345	27581410562	99	2757795975	99
**hopAP1**	Psyr_1890	PsyrB_09325		27581434622[2]	98	2757794721	99
**hopAO1**	-	-	-	-		-	
**hopAO2**	-	-	-	-		-	
**hopAQ1**	-	-	-	-		-	
**hopAR1**	-	-	-	-		-	
**hopAS1**	-	-	-	-		-	
**hopAT1**	-	-	-	-		-	
**hopAU1**	-	-	-	-		-	
**hopAV1**	-	-	-	-		-	
**hopAW1**	-	-	-	-		-	
**hopAX1**	-	-	PsyrH_18845	-		-	
**hopAY1**	Psyr_2631	-	-	2758143760(6)	95	-	
**hopAZ1**	-	-	PsyrH_17410	-		-	
**hopBA1**	-	-	PsyrH_12900	-		-	
**hopBB1**	-	-	-	-		-	
**hopBC1**	-	PsyrB_08275	PsyrH_18105	2758142866(2)	99	-	
**hopBD1**	-	-	-	-		-	
**hopBD2**	-	-	-	-		-	
**hopBE1**	-	-	-	2758144790(5)	98	2757799454(5)	99
**hopBF1**	-	-	-	-		-	
**hopBG1**	-	-	-	-		-	
**hopBH1**	-	-	-	-		-	
**hopBI1**	-	-	-	-		-	
**hopBJ1**	-	-	-	-		-	
**hopBK1**	-	-	PsyrH_113064	-		-	
**hopBL1**	-	-	-	-		-	
**hopBL2**	-	-	-	-		-	
**hopBM1**	-	-	-	-		-	
**hopBN**	-	-	-	-		-	
**Total**	24/97	19/97	26/97	21/97		20/97	

[1] % ID is compared to the Psyr gene from *Pseudomonas sp.* B728a unless otherwise noted.
[2] gene appears truncated
[3] % ID is compared to the PsyrB gene from *Pseudomonas sp*. B301D
[4] % ID is compared to the PsyrH gene from *Pseudomonas sp.* H191
[5] % ID is compared to the hopBE1 gene from *Pseudomonas sp. aceris* MAFF302273
[6] % ID is compared to the hopAY1 gene from *Pseudomonas syringae* sp. *mori* 301020

Table S3. GC content of effector proteins compared to the GC content of the genomes of *P. syringae* NP10-3 and *P. syringae* NP28-5.

Effector	NP10-3		NP28-5	
	gene %GC	%GC vs. genome[1]	gene %GC	%GC vs. genome[1]
**hopH1**	47.6%	10.89%	47.6%	10.93%
**hopBE1**	48.7%	9.76%	48.7%	9.80%
**hopAF1**	-	-	49.0%	9.49%
**hopAL1**	-	-	49.2%	9.29%
**hopBC1**	50.6%	7.87%	-	-
**hopAP1**	51.7%	6.80%	53.7%	4.94%
**hopAY1**	52.1%	6.41%	-	-
**hopAN1**	65.3%	4.87%	65.2%	4.76%
**hopA2**	53.3%	5.28%	-	-
**hopAG1**	53.6%	5.00%	53.6%	5.03%
**hopAJ2**	62.3%	2.52%	61.9%	2.16%
**hopAA1**	62.2%	2.44%	62.1%	2.32%
**hopM1**	62.1%	2.36%	62.1%	2.32%
**hopAC1**	62.0%	2.28%	62.0%	2.24%
**hopW1**	61.9%	2.20%	61.9%	2.16%
**hopAE1**	61.9%	2.20%	61.9%	2.16%
**hopAH2**	61.1%	1.55%	54.9%	3.84%
**hopAH1**	57.7%	1.32%	57.8%	1.26%
**hopI1**	58.0%	1.06%	58.1%	1.01%
**avrE1**	60.4%	0.97%	60.4%	0.94%
**hopAI1**	60.3%	0.89%	60.1%	0.69%
**hopJ1**	60.2%	0.80%	60.8%	1.27%
**hopAK1**	58.5%	0.63%	58.3%	0.83%

[1]%GC vs. genome is calculated as |(gene GC) – (genome GC)|/((gene GC) + (genome GC))

Table S4. Antibiotic susceptibility of *P. syringae* poplar isolates compared to *P. syringae* pv. *tomato* DC3000.

**Antibiotic**	**Resistance gene**	**DC3000**	**NP10-3**	**NP28-5**
Ampicillin	ampC	Resistant	Resistant	Resistant
Apramycin	aac(3) IV	Resistant	Resistant	Resistant
Cefotaxime		Susceptible	Susceptible	Susceptible
Chloramphenicol		Susceptible	Susceptible	Susceptible
Gentamicin		Susceptible	Susceptible	Susceptible
Kanamycin		Susceptible	Susceptible	Susceptible
Nalidixic Acid		Susceptible	Susceptible	Susceptible
Penicillin	ampC	Resistant	Resistant	Resistant
Rifampicin	rpoB*	Resistant	Susceptible	Susceptible
Tetracycline		Susceptible	Susceptible	Susceptible

*mutation confers resistance

Table S5. Physiological effects on *Populus trichocarpa* rooted seedlings after 15 d exposure to *Pseudomonas syringae* isolates.

treatment	final plant mass (g)	final root mass (g)	Δplant mass (g)	stem length (cm)	leaf area (cm^2)	leaf lesion coverage (% total leaf surface affected)	Δ chlorophyll (μmol/m^2 leaf)
Control	0.77 (0.32)	1.04 (0.02)	0.77 (0.32)	11.13 (2.75)	20.68 (7.90)	0.04 (0.01)	-11.33 (4.54)
DC3000	0.84 (0.15)	1.26 (0.08)	0.84 (0.15)	8.32 (3.07)	24.67 (4.43)	0.71 (0.5)	-11.28 (2.74)
NP10-3	0.72 (0.18)	0.41 (0.07)*	0.72 (0.18)	8.45 (3.08)	22.23 (6.30)	2.07 (1.97)	-10.25 (7.37)
NP28-5	0.87 (0.25)	0.35 (0.03)*	0.87 (0.25)	9.17 (1.70)	22.89 (5.66)	6.30 (3.21)*	-6.53 (5.19)

*P<0.05 compared to control

Table S6. Metabolite profile of control and *Pseudomonas*-infected *Populus trichocarpa* rooted plants. Red text indicates metabolite fold change (FC) is positive and the change is significant (Student's t-test reported as P-value); blue text indicates metabolite FC compared to 0 h is decreased and the change is significant.

Populus Metabolite[1]	Control				NP10-3				NP28-5			
	24 h[3]		48 h		24 h		48 h		24 h		48 h	
	FC	p-value	FC	p-value	FC	p-value	FC	p-value	FC	p-value	FC	p-value
catechol glucoside	11.46	0.035	13.80	0.105	17.03	0.035	21.18	0.068	59.72	0.120	32.47	0.114
threonine	1.74	0.565	0.73	0.616	0.93	0.630	0.25	0.138	6.48	0.293	4.67	0.288
salicyl alcohol	2.28	0.358	1.32	0.242	1.44	0.032	3.03	0.183	29.38	0.217	4.21	0.144
catechin	0.86	0.793	2.49	0.190	8.26	0.149	5.28	0.133	1.38	0.615	4.02	0.017
digalactosylglycerol	1.75	0.156	1.08	0.868	4.26	0.009	2.57	0.033	5.57	0.049	3.92	0.003
salicortin	1.28	0.406	1.23	0.634	2.43	0.002	2.35	0.014	2.96	0.036	3.75	0.001
13.82 [169; alcohol glycoside]	1.84	0.022	1.88	0.069	2.83	0.003	3.40	0.009	3.50	0.011	3.64	<0.0001
13.68 [171, 289; glycoside]	1.59	0.063	1.69	0.107	2.69	0.009	2.83	0.002	3.05	0.027	3.56	<0.0001
α-salicyloylsalicin	1.09	0.790	1.24	0.641	2.17	0.027	2.24	0.014	2.95	0.059	3.55	0.004
salicylic acid glucoside	1.91	0.152	1.87	0.020	2.16	0.084	2.67	0.070	2.22	0.191	3.38	0.002
isosalicin	1.97	0.024	2.73	0.046	2.13	0.008	2.76	0.062	5.42	0.148	3.29	0.076
17.68 [476, 386, 296]	1.28	0.552	1.47	0.466	3.15	0.018	2.43	0.046	2.38	0.082	3.06	0.001
citric acid	0.65	0.498	0.79	0.696	3.21	0.126	2.07	0.240	2.33	0.155	2.95	0.082
13.75 [171, 289; glycoside]	1.56	0.078	1.59	0.148	2.35	0.023	2.62	0.010	2.56	0.044	2.83	0.001
2,5-dihydroxybenzoic acid-5-O-glucoside	2.34	0.102	2.20	0.040	6.66	0.011	5.04	0.006	4.87	0.056	2.77	0.029
myo-inositol	1.31	0.423	1.12	0.759	2.38	0.034	2.32	0.042	2.20	0.042	2.72	0.008
fructose	2.95	0.004	2.38	0.104	2.08	0.001	2.20	0.002	2.86	0.004	2.71	0.049
o-cresol-glycoside[2]	1.27	0.387	1.38	0.275	1.43	0.146	1.39	0.088	2.23	0.065	2.42	<0.0001
salicyl-salicylic acid-2-O-glucoside	2.37	0.080	3.12	0.102	1.80	0.235	2.15	0.259	2.87	0.019	2.40	0.045
6-hydroxy-2-cyclohexenone alcohol	0.83	0.696	0.82	0.700	1.36	0.196	0.98	0.901	1.51	0.362	2.35	0.063
6-hydroxy-2-cyclohexenone-1-carboxylic acid	0.74	0.066	0.74	0.203	1.29	0.233	1.06	0.759	1.87	0.168	2.33	0.063
phytol	0.93	0.812	0.29	0.075	1.62	0.177	1.23	0.280	1.40	0.292	2.32	0.089
galactosylglycerol	1.44	0.285	0.95	0.890	2.82	<0.0001	2.38	0.030	4.39	0.088	2.29	0.001
trichocarpin	1.17	0.641	1.17	0.732	1.61	0.089	1.69	0.051	1.74	0.087	2.22	0.010
glyceric acid	3.69	0.024	3.05	0.097	1.56	0.441	1.47	0.038	1.19	0.497	2.22	0.265
10.83 [393, 303, 257]	1.76	0.024	1.91	0.002	1.55	0.024	1.82	0.007	1.85	0.020	2.21	0.006
o-cresol[2]	1.53	0.121	1.87	0.046	1.28	0.212	1.26	0.193	1.89	0.023	1.97	0.055
12.85 [235; glycoside]	1.07	0.869	0.79	0.591	2.70	0.114	2.66	0.017	1.05	0.930	1.92	0.161
aspartic acid	1.57	0.142	1.13	0.323	1.22	0.243	1.02	0.887	2.23	0.179	1.79	0.246
shikimic acid	0.92	0.838	0.91	0.843	1.38	0.125	1.13	0.605	1.45	0.016	1.79	0.230
quercetin	1.11	0.823	0.96	0.932	3.67	0.165	2.50	0.339	1.36	0.373	1.77	0.264
glucose	2.18	0.035	1.85	0.268	1.10	0.970	0.95	0.865	2.07	0.024	1.71	0.065
threonic acid	1.08	0.800	0.79	0.607	1.92	0.016	1.00	0.987	2.37	0.087	1.69	0.159
quinic acid	1.10	0.815	0.54	0.336	2.44	0.031	1.42	0.423	2.32	0.055	1.57	0.349
5-oxo-proline	1.34	0.202	1.02	0.890	0.98	0.768	0.99	0.955	1.38	0.204	1.52	0.131
kaempferol	1.19	0.586	0.96	0.912	2.67	0.315	1.91	0.388	1.17	0.372	1.51	0.334
benzyl alcohol	1.00	0.954	0.92	0.512	0.93	0.924	1.28	0.046	1.33	0.209	1.43	0.136
sucrose	1.62	0.008	1.59	0.003	1.16	0.059	1.31	0.010	1.33	0.071	1.41	0.022
succinic acid	1.18	0.589	0.98	0.946	1.10	0.193	1.07	0.732	1.68	0.065	1.40	0.139
salicylic acid	1.24	0.149	1.36	0.156	0.84	0.275	0.89	0.475	1.68	0.127	1.36	0.128
1,2,4-benzenetriol	1.07	0.739	1.10	0.701	1.73	0.230	1.24	0.543	1.03	0.872	1.35	0.191
erythronic acid	1.82	0.040	1.31	0.373	1.31	0.112	1.04	0.844	1.15	0.185	1.33	0.455
glycerol	1.43	0.077	1.15	0.319	0.96	0.634	1.23	0.348	2.14	0.123	1.33	0.200
β-sitosterol	1.44	0.013	1.40	0.027	1.19	0.086	1.10	0.108	1.16	0.202	1.32	0.035
alphα-tocopherol	1.65	0.025	1.64	0.079	1.45	<0.0001	1.27	0.020	1.35	0.103	1.27	0.311
galactose	2.09	0.226	2.23	0.241	0.53	0.069	0.59	0.166	1.41	0.302	1.22	0.493
caffeic acid	1.18	0.291	1.16	0.390	1.62	0.076	1.22	0.172	1.40	0.272	1.20	0.309
stearic acid	1.38	0.032	1.29	0.029	0.82	0.033	0.84	0.051	1.24	0.056	1.19	0.041
salicin	1.22	0.212	1.18	0.361	1.36	0.128	1.29	0.250	1.08	0.630	1.17	0.332
glutamic acid	1.20	0.677	0.69	0.409	1.53	0.296	0.90	0.770	1.98	0.314	1.15	0.743
myo-Inositol 1-phosphate	1.70	0.113	1.33	0.373	0.76	0.039	0.82	0.191	1.68	0.041	1.14	0.720
6-hydroxy-2-cyclohexenone (enol)	1.19	0.018	0.98	0.830	1.13	0.498	1.14	0.272	1.26	0.102	1.13	0.188
8.62 [235, 324, 309]	1.21	0.047	1.04	0.649	1.18	0.410	1.27	0.057	1.20	0.124	1.10	0.400
14.50 [254, 289, 268; glycoside]	1.23	0.359	0.99	0.966	1.57	0.016	1.30	0.019	1.10	0.603	1.10	0.506
1-o-p-coumaric acid glucoside	1.18	0.265	1.12	0.502	1.31	0.135	1.28	0.219	1.05	0.700	1.09	0.541
alanine	0.88	0.751	0.72	0.188	0.78	0.113	0.62	0.058	2.08	0.145	1.02	0.954
malic acid	1.46	0.109	1.51	0.251	0.89	0.293	0.94	0.378	0.71	0.297	1.01	0.959
catechol	0.61	0.163	0.84	0.561	1.59	0.407	1.19	0.648	1.41	0.430	0.95	0.798
oxalic acid	1.13	0.777	0.89	0.788	0.34	0.006	0.45	0.045	1.50	0.646	0.75	0.544
adenosine	1.38	0.340	1.12	0.753	1.45	0.206	1.18	0.485	1.13	0.597	0.72	0.169
asparagine	0.75	0.761	0.33	0.274	0.63	0.367	0.23	0.141	1.33	0.656	0.61	0.158
mucic acid	1.08	0.819	0.69	0.215	1.27	0.261	1.04	0.736	1.06	0.891	0.60	0.344
ethyl phosphate	1.43	0.350	1.29	0.526	0.59	0.076	0.72	0.267	0.87	0.779	0.59	0.176
phosphate	1.52	0.420	1.23	0.640	0.62	0.114	0.69	0.211	1.05	0.930	0.56	0.239

11.74 [232, 449, 464, 347]	0.69	0.685	0.47	0.370	0.35	0.154	0.20	0.119	0.43	0.107	0.41	0.057

[1] unidentified metabolite identified by retention time (min) and [key m/z; possible metabolite type]
[2] metabolite remains to be confirmed
[3] time point FC is compared to same treatment at 0 h

Supplemental References

1. Collmer A, Badel JL, Charkowski AO, Deng W-L, Fouts DE, Ramos AR, et al. *Pseudomonas syringae* Hrp type III secretion system and effector proteins. Proceedings of the National Academy of Sciences of the United States of America. 2000;97(16):8770.
2. Baltrus DA, Nishimura MT, Romanchuk A, Chang JH, Mukhtar MS, Cherkis K, et al. Dynamic evolution of pathogenicity revealed by sequencing and comparative genomics of 19 *Pseudomonas syringae* isolates. PLoS Pathogens. 2011;7(7):e1002132.
